# Supplementary material for: Does peer education go beyond giving reproductive health information? Cohort study in Bulawayo and Mount Darwin, Zimbabwe
Source: BMJ Open. 2020 Mar 8;10(3):e034436. doi: 10.1136/bmjopen-2019-034436 (PMC7064124; doi:10.1136/bmjopen-2019-034436)
Supplement: Author's manuscript [file bmjopen-2019-034436.draft_revisions.pdf]

BMJ Open is committed to open peer review. As part of this commitment we make the peer review history of every article we publish publicly available.

When an article is published we post the peer reviewers' comments and the authors' responses online. We also post the versions of the paper that were used during peer review. These are the versions that the peer review comments apply to.

The versions of the paper that follow are the versions that were submitted during the peer review process. They are not the versions of record or the final published versions. They should not be cited or distributed as the published version of this manuscript.

BMJ Open is an open access journal and the full, final, typeset and author-corrected version of record of the manuscript is available on our site with no access controls, subscription charges or pay-per-view fees (http://bmjopen.bmj.com).

If you have any questions on BMJ Open's open peer review process please email info.bmjopen@bmj.com

BMJ Open

Does peer education go beyond giving reproductive health information? A cohort study in Bulawayo and Mount Darwin, Zimbabwe

Journal:	*BMJ Open*
Manuscript ID	bmjopen-2019-034436
Article Type:	Original research
Date Submitted by the Author:	19-Sep-2019
Complete List of Authors:	Mangombe, Aveneni; Ministry of Health and Child Care, Family Health<br>Owiti, Philip; International Union Against Tuberculosis and Lung Disease (The Union); National Tuberculosis, Leprosy and Lung Disease Program<br>Madzima, Bernard; Ministry of Health and Child Care<br>Xaba, Sinokuthemba; Ministry of Health and Child Care, Harare Zimbabwe<br>Makoni, Talent; Ministry of Health and Child Care, AIDS and TB Unit ; World Health Organization , Zimbabwe, HIV Prevention<br>Takarinda, Kudakwashe ; Ministry of Health and Child Care<br>Timire, Collins ; International Union Against Tuberculosis and Lung Disease (The Union)<br>Chimwaza, Anesu; Ministry of Health and Child Care<br>Senkoro, Mbazi; National Institute for Medical Research – Muhimbili Centre<br>Mabaya, Simbarashe; World Health Organization Regional Office for Africa<br>Ameyan, Wole ; World Health Organization<br>Samuelson, Julia; World Health Organization<br>Tapera, Talent ; Africaid<br>Zwangobani, Nonhlahla; Zimbabwe National Family Planning Council, Health<br>Tripathy, Jaya; PGIMER, Community Medicine<br>Kumar, Ajay; International Union Against Tuberculosis and Lung Disease (The Union), South East Asia Office, Operational Research; International Union Against Tuberculosis and Lung Disease (The Union), Paris, France
Keywords:	Adolescents, Young people, voluntary medical male circumcision, adolescent sexual and reproductive health, SORT IT

SCHOLARONE™ Manuscripts

I, the Submitting Author has the right to grant and does grant on behalf of all authors of the Work (as defined in the below author licence), an exclusive licence and/or a non-exclusive licence for contributions from authors who are: i) UK Crown employees; ii) where BMJ has agreed a CC-BY licence shall apply, and/or iii) in accordance with the terms applicable for US Federal Government officers or employees acting as part of their official duties; on a worldwide, perpetual, irrevocable, royalty-free basis to BMJ Publishing Group Ltd ("BMJ") its licensees and where the relevant Journal is co-owned by BMJ to the co-owners of the Journal, to publish the Work in this journal and any other BMJ products and to exploit all rights, as set out in our licence.

The Submitting Author accepts and understands that any supply made under these terms is made by BMJ to the Submitting Author unless you are acting as an employee on behalf of your employer or a postgraduate student of an affiliated institution which is paying any applicable article publishing charge ("APC") for Open Access articles. Where the Submitting Author wishes to make the Work available on an Open Access basis (and intends to pay the relevant APC), the terms of reuse of such Open Access shall be governed by a Creative Commons licence – details of these licences and which Creative Commons licence will apply to this Work are set out in our licence referred to above.

Other than as permitted in any relevant BMJ Author's Self Archiving Policies, I confirm this Work has not been accepted for publication elsewhere, is not being considered for publication elsewhere and does not duplicate material already published. I confirm all authors consent to publication of this Work and authorise the granting of this licence.

For peer review only

Title: Does peer education go beyond giving reproductive health information? A cohort study in Bulawayo and Mount Darwin, Zimbabwe

Manuscript type: Original research article

Short Running Title: Enhanced reproductive health service uptake through peer education in Zimbabwe

Investigators and Affiliations

Aveneni Mangombe[1], Philip Owiti[2,3], Bernard Madzima[1], Sinokuthemba Xaba[1], Talent Makoni[1], Kudakwashe Collin Takarinda[1,2], Collins Timire[1,2], Anesu Chimwaza[1], Mbazi Senkoro[4], Simbarashe Mabaya[5], Julia Samuelson[6], Wole Ameyan[6], Talent Tapera[7], Nonhlanhla Zwangobani[8], Jaya Prasad Tripathy[9,10], Ajay MV Kumar[2,9,11]

1. Ministry of Health and Child Care, Harare, Zimbabwe
2. International Union Against Tuberculosis and Lung Disease (The Union), Paris, France;
3. National Tuberculosis, Leprosy and Lung Disease Program, Nairobi, Kenya
4. National Institute for Medical Research – Muhimbili Centre, Dar es Salaam, Tanzania
5. World Health Organization, Harare. Zimbabwe
6. World Health Organization, Geneva, Switzerland
7. Africaid, Harare, Zimbabwe
8. Zimbabwe National Family Planning Council, Harare, Zimbabwe
9. International Union Against Tuberculosis and Lung Disease, South-East Asia Office, New Delhi, India
10. All India Institute of Medical Sciences, Nagpur, India
11. Yenepoya Medical College, Yenepoya (Deemed to be University), Mangaluru, India

For peer review only - http://bmjopen.bmj.com/site/about/guidelines.xhtml

Corresponding Author:

Aveneni Mangombe, National Adolescent and Youth Sexual Reproductive Health Programme Coordinator, Ministry of Health and Child Care, Harare, Zimbabwe.

Email: mangombeaveh@gmail.com Telephone: +263773302742

Manuscript details

Word count: Abstract: 300 words; Main text: 3575

Number of tables: 6; **Number of figures**: 0

Number of references: 25

For peer review only

ABSTRACT

Objective: Peer education has largely been a community-based intervention within the voluntary medical male circumcision (VMMC) – adolescent sexual and reproductive health (ASRH) linkages pilot project in Bulawayo and Mount Darwin, Zimbabwe since 2016. However, little is known if benefits extend beyond information-giving. We assessed the extent of and factors affecting referral and receipt of HIV testing services (HTS), contraception, diagnosis and treatment of sexually transmitted infections (STIs) and VMMC services by young people (10 – 24 years) counselled by peer educators in the two project areas during October – December 2018.

Design: A cohort study utilizing the project's routinely collected secondary data.

Setting: All the ASRH and VMMC sites in Mt Darwin and Bulawayo.

Participants: 95 peer educators.

Primary outcome measures: The censor date for assessing receipt of services was 31 January 2019. Factors affecting non-referral and non-receipt of services were assessed by log-binomial regression. Adjusted risk ratios (aRR) were calculated.

Results: Of the 3370 counseled, 65% were referred for at-least one service. 58% of the counseled males were referred for VMMC. Other services had 5-13% referrals. Non-referral for HTS decreased with clients' age(aRR:~0.9) but higher among group-counselled than individually-counselled (aRR:1.16). Counseling by male peers(aRR:0.77) and rural location(aRR:0.61) reduced the risks of non-referral for VMMC while age increased it(aRR≥1.59). Receipt of services was high at 64-80% except for those referred for STIs (39%). Group counseling and rural location(aRR:~0.52) and male peer educators(aRR:0.76) reduced risk of non-receipt of VMMC. Rural location increased risk of non-receipt of contraception(aRR:3.18) while marriage reduced it(aRR:0.20).

For peer review only - http://bmjopen.bmj.com/site/about/guidelines.xhtml

Conclusion: This study found moderate levels of referrals with variations by service types but high levels of receipt of services among referred clients. Type of counselling, gender of peer educators and location affected receipt of services. The study recommends qualitative approaches to further understand the reasons for non-referrals and non-receipt of services.

Key words: Adolescents, Young people, ASRH, Sexual and reproductive health, voluntary medical male circumcision, HIV Testing, Contraception, Sexually transmitted infections, Uptake and SORT IT.

Article Summary

Strengths and Limitations of this Study:

- This study included all the clients who were counselled by peer educators during the study period in the two project districts;
- It used routine programme data, thus making the findings a likely true reflection of the situation;
- The conduct and reporting of the study adhered to Strengthening the reporting of observational studies in epidemiology (STROBE) guidelines.
- The study couldn't exclude clients not in need of referral from the analysis due to lack of data from the peer educator registers in determining the reason for non-referral;
- Due to small sample size of those referred for STI diagnosis and treatment, the study lacked statistical power to carry out further analyses.

For peer review only - http://bmjopen.bmj.com/site/about/guidelines.xhtml

INTRODUCTION

Human Immunodeficiency Virus (HIV) still remains a major global public health concern, with 1.8 million new infections and 940,000 deaths reported in 2017.(1) Of concern is the slower decline of Acquired Immune Deficiency Syndrome (AIDS)-related illnesses and deaths among young people (10-24 years) compared to adults.(2) Coverage of HIV testing and access to treatment remain significantly low among young people, especially in sub-Saharan Africa.(2) Studies and systematic reviews have reported high rates of sexually transmitted infections (STIs), teenage pregnancy and suicide attempts, coupled with poor receipt of sexual and reproductive health (SRH) and HIV services among young people.(2,3)

Zimbabwe is a low-income country with a population of nearly 13.5 million as of 2017.(4) A third of the country's population are young people aged 10-24 years.(5) Mirroring the global scenario, the Zimbabwe Demographic Health Survey (ZDHS) 2015 reported a high fertility rate among young girls (15-19 years), low coverage of HIV testing among 15-19 years old (35%-46%) and low comprehensive knowledge on HIV.(6) Though SRH and HIV interventions focused on young people have been in existence for the past three decades, in Zimbabwe, the first 5-year strategic plan was developed in 2010. Among other things, this phase defined a minimum package of interventions that recognised the role of community-based youth peer educators in educating young people on SRH and HIV.

There is no universally agreed upon definition of peer education. We define it as a structured process of sharing of relevant information, values and behaviours among members of similar status, in an appropriate setting for both the educator and learner. Following a series of reviews, peer education has been noted to be an effective youth-led approach for influencing positive behavioural outcomes among beneficiaries, if given appropriate support systems and contextualized to different settings and needs of beneficiaries.(7,8) Peer education has been

For peer review only - http://bmjopen.bmj.com/site/about/guidelines.xhtml

established to be more effective in reaching out to key populations (for example, adolescents selling sex and who are sexually exploited) and delivering messages that are considered taboo to be delivered through schools, religious and family settings.(9) Integration of peer education interventions with holistic and well-coordinated interventions make it more effective towards improving health outcomes among beneficiaries in different contexts.(10,11)

In 2016, a project to link Voluntary Medical Male Circumcision (VMMC) with the Adolescent Sexual and Reproductive Health (ASRH) services (*"Smart LyncAges Project")* was started. In 2017, the scope of this project was expanded to engage youth peer educators in promoting referral and receipt of SRH and VMMC services in Bulawayo and Mount Darwin districts. Informed by Michielsen et al's review(12), the re-designing incorporated provision of an updated training that introduced relevant tools for delivering the messages (distribution of information, education and communication materials and social media platforms such as facebook and WhatsApp), which defined a clear referral pathway (including referral forms, tracking and two-way feedback mechanism) and provision of adequate tools for documentation.

Since the re-designing of the peer education component, no study has been conducted to determine the effect of peer educators beyond just providing information to their peers. Within this background, we carried out a study to assess the referral and receipt of HIV testing services (HTS), contraception, diagnosis and treatment of sexually transmitted infections (STIs) and VMMC services among young people (10 – 24 years) counselled by the peer educators and their associated factors within the two pilot districts (Bulawayo and Mount Darwin) in Zimbabwe.

For peer review only - http://bmjopen.bmj.com/site/about/guidelines.xhtml

METHODS

Study design:

This was a cohort study involving secondary analysis of routinely collected data under the VMMC - ASRH linkages pilot project in Zimbabwe.

Setting:

General setting

In 2009, Zimbabwe devised the National ASRH strategy 2010-15 to promote adoption of safer SRH practices and increase availability, access and utilization of SRH and HIV services by young people.(13) The strategy outlined three settings for providing 'friendly' SRH and HIV services: health facility, community and school-based. The health facility approach required every facility to establish and equip special rooms (youth-friendly corners). The community approach involved establishment of 'community youth centres (CYCs)' whilst the school-based approach focussed on the provision of life skills education and counselling mainly by teachers.

In 2015, an extensive review of the 2010-2015 interventions in Zimbabwe was conducted so as to inform the development of the National ASRH Strategy II: 2016-2020.(14) One of the review's conclusions acknowledged peer education as an important tool in ASRH programming, though with some modifications.(15) This strategy seeks to strengthen comprehensive sexuality education (CSE) and provision of quality-assured and adolescent-friendly services, delivered through schools and colleges, public health facilities and the community. As part of expanding comprehensive sexuality education in both the in- and out-of-school settings, Zimbabwe aligned its tools and training materials with the 2018 United Nations Educational, Scientific and Cultural Organization (UNESCO) revised international

For peer review only - http://bmjopen.bmj.com/site/about/guidelines.xhtml

technical guidance on sexuality education.(16) The minimum package of SRH services for young people includes contraception, STI diagnosis and treatment, HTS and integration of VMMC.

The VMMC - ASRH *linkages project*

The VMMC - ASRH linkages project has been implemented in Bulawayo and Mount Darwin, Zimbabwe since 2016. Bulawayo is an urban city with 27 public health facilities while Mount Darwin is a rural district with 19 public health facilities. In Mount Darwin, VMMC services are provided at the Mount Darwin Hospital, whilst diagnosis and treatment of STIs, contraception and HTS services are being provided at Mount Darwin Hospital and two CYCs (Mt Darwin and Dotito) supported through the Zimbabwe National Family Planning Council. In Bulawayo, VMMC services are provided at the Bulawayo Male Circumcision (MC) site and the Lobengula MC site and sometimes through outreach camps in the 15 CYCs and clinics. The CYCs in Bulawayo are primarily focused on imparting information and counselling services, edutainment services through films, drama and sports, library services and vocational and life skills training. All the service delivery points under this project were oriented on the VMMC – ASRH linkages service delivery protocols and ASRH. The VMMC – ASRH service delivery protocols clearly highlight the scope of work, reporting, referral, coordination and supervision mechanisms for peer educators. Demand creation for both SRH and VMMC services for young people relies heavily on community-based peer educators and VMMC mobilisers.

Peer education

Each CYC is expected to coordinate and supervise the work of 5 – 6 peer educators at any point in time. Peer educators are volunteers aged 10-24 years, residing in the community and nominated by the young people in stakeholder community meetings. They must be able to

For peer review only - http://bmjopen.bmj.com/site/about/guidelines.xhtml

read and write in English and the respective local language and should have passed at least 3 subjects at the end of secondary level (13th grade). Under the *Smart LyncAges project*, peer educators undergo a seven-day standard training, which also addresses the referral pathway. They are attached to the nearest CYC and are allocated a catchment area to cover. Peer educators are expected to contribute at least two hours a day for at least 3 days in a week to the project. Active peer educators receive a fixed monthly allowance of 15 USD, paid upon submission of daily and monthly summary reports of their activities.

While peer educators spend most of their time in the community conducting outreach sessions, they do sit in CYCs on a rotation basis to cater for the walk-in clients. Peer educators reach clients through both individual *(one-on-one)* and group counselling sessions. They choose places such as recreation parks, play grounds, schools and community halls where young people usually congregate. In addition to providing information and counselling services, peer educators also distribute condoms to the clients. However, they refer clients for such when they run out of stock or are in settings (such as churches and schools) where condom distribution is prohibited.

Peer educators also facilitate referrals to ASRH and VMMC sites. The referral process includes providing information regarding location of the service delivery point, hours of operation, user fees (if any) and details of contact person (if available). They also complete referral forms in triplicate (one copy kept for records and two copies sent with the client to the destination service delivery point). One copy of the latter is retained at the service delivery point, while the other is given to the client who is expected to return it to the respective peer educator. The form retained at the service delivery point is sent to the respective CYC by post or hand-delivered. This system facilitates feedback on receipt of services by referred clients. Monthly, the peer educators are expected to track all the referred clients, through verification of redeemed referral forms, physical home visits or by telephone

For peer review only - http://bmjopen.bmj.com/site/about/guidelines.xhtml

(where possible). In some cases, peer educators accompany clients to the service delivery points to ensure that they avail the service.

As part of documentation, each peer educator maintains individualized *"daily and monthly summary peer educator registers'* where they document the socio-demographic details of the clients who received counselling, the referrals and receipt of the services. Each service delivery point under the VMMC - ASRH Linkages project also uses primary registers for SRH and HIV services provision.

<u>Study population:</u>

All young people (10-24 years) who were counselled by youth peer educators during the period October – December 2018 in Bulawayo city and Mount Darwin district were included in the study.

<u>Data variables, sources of data and data collection:</u>

A structured proforma was used to collect the following data variables: socio-demographic characteristics of clients (age, sex, marital status, location, schooling status, and type of counselling sessions undergone), the age and sex of their peer educators, whether referred or not, services referred for and if the clients received the services. The source of data was the peer educator registers. 'Referral' in this study meant that clients consented to receive some post-counseling services (such as HIV testing, VMMC, contraception or STI diagnosis and treatment) and were given a referral form. 'Receipt' of a service meant receiving/getting the services at a referral service delivery point by the young person who had been referred by peer educators (between October and December 2018) by end of January 2019.

<u>Data Analysis and statistics:</u>

For peer review only - http://bmjopen.bmj.com/site/about/guidelines.xhtml

Data entry and validation was performed using EpiData software (*v4.4.1.0, EpiData Association, Odense, Denmark*) while analyses were carried out using EpiData Analysis (*v2.2.2.186*) and STATA (*v14, StataCorp, College Station, Texas, USA*) softwares. Proportions were used to summarize referrals and receipt of services for those referred. There were four major services for which the clients were referred: HTS, VMMC, contraception and STI diagnosis and treatment. Of these, factors associated with non-referral were assessed for HTS and VMMC services only. Factors associated with non-referrals for contraception and STI diagnosis and treatment were not analyzed as it was not possible to establish the appropriate denominator defining the eligibility for these services. Factors associated with non-receipt of services were assessed for VMMC and contraceptive use only. Non-receipt of HTS was not analyzed as HTS can also be provided as an opt-out provider-initiated testing and counseling (PITC) service at all the service delivery points, irrespective of the reason for which the client was referred. HTS was also a necessary service prior to conducting VMMC. Non-receipt of STI services amongst those referred was not analyzed due to small sample size. The strength of associations were initially expressed using unadjusted relative risks and then further expressed using adjusted relative risks (aRR) and 95% confidence intervals, using log-binomial regression methods. Two-sided $P<0.05$ were considered statistically significant.

For peer review only - http://bmjopen.bmj.com/site/about/guidelines.xhtml

RESULTS

There were 95 peer educators (52% males) in the study sites with a median age of 22 years (range: 15-24). A total of 3370 young people received counselling services from the peer educators (**Table 1**). Forty percent of young people were aged 15-19 years, with the majority being males (66%) and single (98%). Majority were still in school (69%) and received group counselling (78%).

Referrals and receipt of services

Table 2 shows the proportions of clients who were referred and those who received the services among those referred. Sixty five percent of the young people counselled were referred for SRH services. Of those referred, 77% had been referred for only one service, with the rest being referred for two or more services. Majority (75%) of those referred received the services they had been referred for.

Of the males counselled, 58% were referred for VMMC services, of whom, 69% received the services. The other services for which the adolescents were referred for include HIV testing (13%), contraception (13%) and diagnosis and treatment of STIs (5%). Among the services referred for, the receipt for STI services was lowest (39%).

Factors associated with non-referrals for HTS and VMMC

Non-referral for HIV Testing Services (**Table 3**)

In multivariable analysis, only age of client and type of counseling were significantly associated with non-referral for HTS. Adolescents aged 15-19 years had a 9% (95% CI: 0.81-1.03) reduced risk of non-referral for HTS as compared to those aged 10-14 years old. Those who underwent group counseling had 16% (95% CI: 1.04-1.27) increased risk of non-referral for HTS. Sex and marital status of the peer educators did not influence referral for HTS.

For peer review only

For peer review only - http://bmjopen.bmj.com/site/about/guidelines.xhtml

Non-referral for VMMC services (**Table 4**)

Compared to clients in the 10-14 year age group, those in the 15-19 year and 20-24 year age group had 59% (95% CI: 1.34-1.87) and 83% (95% CI: 1.49-2.26) increased risk of non-referral for VMMC, respectively. Clients in the rural district of Mount Darwin were at 39% (95% CI: 0.52-0.71) reduced risk of non-referral compared to those in the urban city of Bulawayo. Clients counseled on VMMC by male peer educators were at 23% (95% CI: 0.67-0.80) reduced risk for non-referral as compared to those counselled by female peer educators.

Factors associated with non-receipt for VMMC and contraception

Non-receipt of VMMC services (**Table 5**)

Clients referred by male peer educators had a 24% (95% CI: 0.62-0.94) lower risk of non-receipt of services than those referred by their female counterparts. Those referred from the rural district of Mount Darwin (aRR: 0.52, 95% CI: 0.41-0.67) and those referred through group counselling sessions (aRR: 0.52, 95% CI: 0.41-0.66) had significantly lower risk of non-receipt of VMMC services as compared to urban Bulawayo and individual counseling sessions, respectively. Age of the clients did not influence receipt of VMMC services.

Non-receipt of contraception services (**Table 6)**

Clients referred from the rural district had 3.2 times (95% CI: 1.93-5.22) higher risk of non-receipt of contraception services as compared to the urban-based counterparts. Married clients had 80% (95% CI: 0.07-0.58) reduced risk of not receiving contraception services than the single clients.

For peer review only - http://bmjopen.bmj.com/site/about/guidelines.xhtml

DISCUSSION

This study, the first one assessing the role of the peer educators in the referral and receipt of selected SRH and VMMC services among young people in Zimbabwe found relatively moderate levels of referrals, particularly for VMMC services. However, for those referred, receipt of the services was high except for STI diagnosis and treatment. Majority of the referred clients had been referred for just one service, perhaps indicating limited counselling or reflecting the lack of need. The risk of not being referred for HTS services decreased slightly with older age of clients, though for VMMC services younger aged adolescents were more likely to be referred. Slightly increased non-referral for HTS services was observed in clients who underwent group counseling sessions as compared to individual sessions. With regard to VMMC services, those counseled by male peer educators were more likely to be referred and to receive, likewise to the clients in the rural district. Rural setting increased risk of not receiving contraception services while being married reduced this risk.

The low referral for diagnosis and treatment of STIs might have been influenced by: 1) lack of confidence by peer educators in determining the need for referral. 2) limited self-reports of STI-like symptoms. In any population, only a minority will have STI or STI - like symptoms and referrals rates may be low. However, the low receipt of the diagnosis and treatment of STIs services among the referred clients might have been due to the associated user fees levied (mostly in council clinics and hospitals) in both the rural and urban settings.(17),(18) Abolishing or largely subsidizing these costs, while maintaining or sustaining supply and diagnostics may improve receipt of services by young people.

Low risk perception among early adolescents has been established to be one of the barriers for referral and receipt of HTS services.(19) Other studies have also shown that demand for HTS services are reduced in adolescents who require parental/guardian consent, especially

For peer review only - http://bmjopen.bmj.com/site/about/guidelines.xhtml

due to the perceived negative reactions from parents/guardians.(19) With Zimbabwe's age of consent for HTS at 16 years, this may explain the reduced non-referral for HTS as the client's age increases. Individualized or client-centered counseling has been established to be a more effective approach for HTS and post-test services than group counseling,(20) as demonstrated also by this study.

Group counseling sessions may have provided an opportunity for client peer influence on receipt of VMMC services. Counseling by male peer educators may also encourage openness among the male clients leading to more referral and receipt of VMMC services. As regards contraception, urban areas present more convenient service delivery points for accessing contraceptives than rural areas, explaining why non-receipt is lower in the urban setups. Myths on the association between modern contraception and future infertility(21),(22) may have resulted in the higher non-receipt rates of contraception among the adolescent girls referred. This belief may be more common in the rural setups and at times is propagated by other health workers.(22),(17)

Strengths: This study had several strengths. We included all the clients who were counselled by peer educators during the study period in the two project districts, which were the only districts implementing this intervention in the whole country. As we used routine programme data, the findings are likely a true reflection of the situation. The data was extracted into standard proformas by the principal investigator and trained data collectors – this enhanced quality. Lastly, the conduct and reporting of the study adhered to Strengthening the Reporting of observational studies in epidemiology (STROBE) cohort reporting guidelines.(23)

Limitations: However, there were also limitations. First, due to deficiencies in documentation in the peer educator registers, we could not determine if the reason for non-

For peer review only - http://bmjopen.bmj.com/site/about/guidelines.xhtml

338 referral (for VMMC or HTS) was related to 'need', that is, they had already received VMMC
339 or HTS services before. We, therefore couldn't exclude clients not in need from the analysis.
340 In certain circumstances, like referral for contraception and STI diagnosis and treatment, we
341 could not perform further analyses as it was not possible to establish the appropriate
342 denominator defining the eligibility for these services. Second, we were not able to establish
343 exact reasons for non-referral and non-receipt of services. This requires qualitative and
344 youth-led study approaches. Third, due to small sample size of those referred for STI
345 diagnosis and treatment, we lacked statistical power to carry out further analyses. Many
346 reviews on peer education have largely focused on assessing the designs or models of peer
347 education without focusing on the outputs and the immediate positive outcomes of peer
348 education in relation to receipt of diagnosis and treatment of STIs, HTS, contraception and
349 VMMC services. Therefore, there were no study results to compare with. Whilst the results
350 and conclusions of this study may be used in different peer education interventions, they
351 cannot be generalised.

352 **Implications:** The study has the following implications:

353 • There is need for a review of peer education data collection tools to capture more
354 client and peer educator data, for example, on details and quality of sessions and
355 eligibility of clients for the various services in line with the recently adapted revised
356 international guidelines on CSE in Zimbabwe.(24) The review will also need to be
357 followed with refresher training on the new concepts. This will help in determining
358 actual output and factors associated with it.

359 • The project needs to consider integrating parent-child communication interventions
360 into the VMMC – ASRH linkages project so as to mobilize parents to support the
361 young people to access services (both psychosocially and financially). In the long run,

For peer review only - http://bmjopen.bmj.com/site/about/guidelines.xhtml

this may also provide opportunities for home-based HTS and supervised HIV self-testing.

- While HTS services are better provided through individual sessions, VMMC services are better received when group counseling sessions are provided. There is thus need to focus on differentiated service provisions depending on circumstances, even as services are integrated.
- In view of the male peer educators having higher chances of effectively referring clients for VMMC, the peer education component needs to differentiate approaches which might include pairing of female peer educators with their male counterparts.
- There is a need to review and possibly abolish user fees attached to the diagnosis and treatment of STIs among the adolescents and young people in need of such services.
- Global standards on provision of quality health services to young people should be adopted and client satisfaction surveys prioritized to enhance quality service delivery.(17),(25) This will help contextualize peer education into service delivery and help understand the reasons for non-referral and non-receipt of services.

For peer review only

For peer review only - http://bmjopen.bmj.com/site/about/guidelines.xhtml

CONCLUSION

This operational study found moderate levels of referrals among clients counseled by young peer educators with variation by service type. Receipt of contraception, VMMC and HTS services was high among those referred. Factors affecting non-referral included age of client, sex of peer educator and type of counseling session (individual/group) while type of setting (rural/urban), age of client and sex of peer educators affected non-receipt of services. Peer education service differentiation based on gender and service type may further enhance uptake. There is also a need to review the output of the peer educator project with a view to enhancing the quality of care provided to the adolescents and young people.

For peer review only

For peer review only - http://bmjopen.bmj.com/site/about/guidelines.xhtml

Acknowledgements

This research was conducted through the Structured Operational Research and Training Initiative (SORT IT), a global partnership led by the Special Programme for Research and Training in Tropical Diseases at the World Health Organization (WHO/TDR). The training model is based on a course developed jointly by the International Union Against Tuberculosis and Lung Disease (The Union) and Medécins sans Frontières (MSF). The specific SORT IT program which resulted in this publication was implemented by the Centre for Operational Research, The Union, Paris, France. Mentorship and the coordination/facilitation of this particular SORT IT workshop was provided through the Centre for Operational Research, The Union, Paris, France; the Department of Tuberculosis and HIV, The Union, Paris, France; the University of Washington, School of Public Health, Department of Global Health, Seattle, Washington, USA; National Institute for Medical Research, Muhimbili Centre, Dar es Salaam, Tanzania; and AIDS & TB Department, Ministry of Health & Child Care, Harare, Zimbabwe

Funding

The training course under which this study was conducted was funded by: the United Kingdom's Department for International Development (DFID); The Global Fund to Fight AIDS, Tuberculosis and Malaria (GFATM) and the World Health Organization. The funders had no role in study design, data collection and analysis, decision to publish, or preparation of the manuscript.

Author contributions

AM – the Principal Investigator

AM, TM, NZ, SM, SX and BM conceived the study.

AM, PO, AK, KCT, CT, JPT, NZ, SM, AC and TT designed the study protocol.

For peer review only - http://bmjopen.bmj.com/site/about/guidelines.xhtml

AM, TM, NZ, SM, SX, BM, JS, WA, JPT and AC read and approved the protocol.

AM, TM collected the data.

AM, PO, AK, KCT, CT, JPT, NZ, SM, AC and TT contributed to analyzing and interpreting the data.

AM, AK, PO and TT drafted the manuscript.

AM, AK, PO, TM, NZ, SM, JS, WA, AC, JPT, SX and BM critically revised the manuscript for intellectual content

All authors read and approved the final manuscript.

Disclosure Statement

The authors report no conflicts of interest

Ethics and Consent

Permission to undertake the study was granted by the Ministry of Health and Child Care. Ethics approval was obtained from the Medical Research Council of Zimbabwe (MRCZ/E/223) and the Ethics Advisory Group of the International Union Against Tuberculosis and Lung Disease, Paris, France (EAG number: 62/18). As this was a retrospective analysis of de-identified routine data, the need for individual client consent was waived by both ethics committees.

Data availability statement

The corresponding author can avail the data set on request without undue reservation.

Patient and Public Involvement

The principal investigator and enumerators had no interaction directly with the adolescents and young people whose records were reviewed. The registers were accessed from peer educators, community youth centre and health facility staff.

For peer review only - http://bmjopen.bmj.com/site/about/guidelines.xhtml

REFERENCES

1. Joint United Nations Programme on HIV/AIDS. Global HIV & AIDS statistics — 2018 fact sheet [Internet]. 2018 [cited 2018 Aug 10]. Available from: http://www.unaids.org/en/resources/fact-sheet

2. UNAIDS. Ending the AIDS epidemic for adolescents, with adolescents A practical guide to meaningfully engage adolescents in the AIDS response [Internet]. 2016 [cited 2019 May 9]. Available from: http://www.unaids.org/sites/default/files/media_asset/ending-AIDS-epidemic-adolescents_en.pdf

3. Denno DM, Hoopes AJ, Chandra-Mouli V. Effective Strategies to Provide Adolescent Sexual and Reproductive Health Services and to Increase Demand and Community Support. J Adolesc Heal [Internet]. 2015 Jan [cited 2018 Aug 10];56(1):S22–41. Available from: http://www.ncbi.nlm.nih.gov/pubmed/25528977

4. Zimbabwe National Statistics Agency. Inter-Censal Demographic Survey, 2017. Harare; 2017.

5. Zimbabwe National Statistics Agency. Zimbabwe Population Census 2012 [Internet]. Harare; 2013. Available from: http://www.zimstat.co.zw/dmdocuments/Census/CensusResults2012/National_Report.pdf

6. Zimbabwe National Statistics Agency and ICF International. Zimbabwe Demographic and Health Survey 2015: Final Report. Rockville, Maryland, USA; 2016.

7. Abdi F, Simbar M. The Peer Education Approach in Adolescents- Narrative Review Article. Iran J Public Health [Internet]. 2013 Nov [cited 2019 May 8];42(11):1200–6. Available from: http://www.ncbi.nlm.nih.gov/pubmed/26171331

8. Campbell C, Scott K, Mupambireyi Z, Nhamo M, Nyamukapa C, Skovdal M, et al. Community resistance to a peer education programme in Zimbabwe. BMC Health Serv Res [Internet]. 2014 Dec 19 [cited 2019 May 8];14(1):574. Available from: https://bmchealthservres.biomedcentral.com/articles/10.1186/s12913-014-0574-5

9. Hutton G, Wyss K, N'Diékhor Y. Prioritization of prevention activities to combat the spread of HIV/AIDS in resource constrained settings: a cost-effectiveness analysis from Chad, Central Africa. Int J Health Plann Manage [Internet]. 2003 Apr [cited 2019 May 9];18(2):117–36. Available from: http://www.ncbi.nlm.nih.gov/pubmed/12841152

10. Medley A, Kennedy C, O'Reilly K, Sweat M. Effectiveness of Peer Education Interventions for HIV Prevention in Developing Countries: A Systematic Review and Meta-Analysis. AIDS Educ Prev [Internet]. 2009 Jun [cited 2018 Aug 9];21(3):181–206. Available from: http://www.ncbi.nlm.nih.gov/pubmed/19519235

11. Chandra-Mouli V, Lane C, Wong S. What Does Not Work in Adolescent Sexual and Reproductive Health: A Review of Evidence on Interventions Commonly Accepted as Best Practices. Glob Heal Sci Pract [Internet]. 2015 Aug 31 [cited 2018 Aug 9];3(3):333–40. Available from: http://www.ghspjournal.org/lookup/doi/10.9745/GHSP-D-15-00126

For peer review only
For peer review only - http://bmjopen.bmj.com/site/about/guidelines.xhtml

12. Michielsen K, Chersich MF, Luchters S, De Koker P, Van Rossem R, Temmerman M. Effectiveness of HIV prevention for youth in sub-Saharan Africa: systematic review and meta-analysis of randomized and nonrandomized trials. AIDS [Internet]. 2010 May [cited 2019 May 8];24(8):1193–202. Available from: https://insights.ovid.com/crossref?an=00002030-201005150-00014

13. Ministry of Health and Child Care Zimbabwe. National Adolescent Sexual and Reproductive Health Strategy: 2010 - 2015. Harare; 2009.

14. Ministry of Health and Child Care Zimbabwe. National Adolescent and Youth Sexual and Reproductive Health (ASRH) Strategy II: 2016 -2020. Harare; 2016.

15. Blum RW, Mmari K, Alfonso NY, Posner E. ASRH Strategic Plan Review for Zimbabwe. Harare, Zimbabwe; 2015.

16. UNESCO. International technical guidance on sexuality education An evidence-informed approach [Internet]. 2018 [cited 2019 May 9]. Available from: http://www.unaids.org/sites/default/files/media_asset/ITGSE_en.pdf

17. Youth Engage. Mystery Client Visits in Zimbabwe Report: June 2017 - July 2018. 2018.

18. Ministry of Health and Child Care Zimbabwe. Zimbabwe Health Financing Strategy. 2017.

19. World Health Organisation. Adolescent HIV testing and counselling: a review of the literature [Internet]. World Health Organization; 2013 [cited 2019 May 8]. Available from: https://www.ncbi.nlm.nih.gov/books/NBK217943/

20. Sheon N. Theory and Practice of Client-Centered Counseling and Testing [Internet]. 2006. 2006 [cited 2019 May 8]. Available from: http://hivinsite.ucsf.edu/InSite?page=kb-07-01-04#S9.1X

21. Remez L, Woog V MM. Sexual and Reproductive Health Needs Of Adolescents in Zimbabwe. Brief, New York, Guttmacher Inst [Internet]. [cited 2019 May 8];2014 Serie(No 3):8. Available from: https://www.guttmacher.org/sites/default/files/report_pdf/ib-zimbabwe_0.pdf

22. Ngome E, Odimegwu C. The social context of adolescent women's use of modern contraceptives in Zimbabwe: a multilevel analysis. Reprod Health [Internet]. 2014 Dec 10 [cited 2019 May 8];11(1):64. Available from: http://www.ncbi.nlm.nih.gov/pubmed/25108444

23. von Elm E, Altman DG, Egger M, Pocock SJ, Gøtzsche PC, Vandenbroucke JP, et al. Strengthening the Reporting of Observational Studies in Epidemiology (STROBE) statement: guidelines for reporting observational studies. Lancet (London, England) [Internet]. 2007 Oct 20 [cited 2019 May 8];370(9596):1453–7. Available from: http://www.ncbi.nlm.nih.gov/pubmed/18064739

24. Herat J, Plesons M, Castle C, Babb J, Chandra-Mouli V. The revised international technical guidance on sexuality education - a powerful tool at an important crossroads for sexuality education [Internet]. Vol. 15, Reproductive Health. BioMed Central; 2018 Dec [cited 2019 May 9]. Available from: https://reproductive-health-journal.biomedcentral.com/articles/10.1186/s12978-018-0629-x

For peer review only

520 25. Ministry of Health and Child Care Zimbabwe. National Guidelines on Clinical Adolescent and Youth Friendly Sexual and Reproductive Health Services Provision (YFSP). 2016.

For peer review only

For peer review only - http://bmjopen.bmj.com/site/about/guidelines.xhtml

Table 1: Socio-demographic characteristics of young people counselled by peer educators in Bulawayo and Mount Darwin, Zimbabwe (October – December 2018):

Characteristics		N	(%)
Total		3370	(100)
Age (years)			
	10 – 14	1242	(36.9)
	15 – 19	1346	(39.9)
	20 – 24	782	(23.2)
Sex			
	Male	2207	(65.5)
	Female	1163	(34.5)
Marital Status			
	Single	3288	(97.6)
	Married	77	(2.3)
	Divorced/Separated	5	(0.1)
Schooling status			
	In-school	2334	(69.3)
	Out of school	1036	(30.7)
Type of counseling session received			
	Individual	741	(22.0)
	Group	2629	(78.0)
Type of Setting			
	Bulawayo (Urban)	2160	(64.1)
	Mount Darwin (Rural)	1210	(35.9)
Age of peer educator (years) [a]			
	15 - 19	751	(22.3)
	20 - 24	2619	(77.7)
Sex of peer educator [b]			
	Male	1624	(48.2)
	Female	1746	(51.8)

[a] Refers to the number of clients who were counselled by the peer educators aged 15 – 19 and 20 - 24 years.

[b] Refers to the number of clients who were counselled by male and female peer educators.

For peer review only

For peer review only - http://bmjopen.bmj.com/site/about/guidelines.xhtml

Table 2: Services for which counselled young people were referred for by peer educators and received in Bulawayo and Mount Darwin districts, Zimbabwe (October – December 2018)

**Type of Service**	**Referred****		**Received****	
	**n**	**(%)**[a]	**n**	**(%)**[b]
Referred for any service	2191	(65.0)	1645	(75.1)
HTS	424	(12.6)	271	(63.9)
VMMC *(among males only; n=2207)*	1287	(58.3)	881	(68.5)
Contraception*	452	(13.4)	363	(80.3)
STI diagnosis and treatment	171	(5.1)	67	(39.2)
Other SRH and HIV service	497	(14.7)	397	(79.9)

[a] – denominator is the total number counselled (N=3370), except for VMMC

[b] – denominator is the total number referred in the respective category

*Contraception refers to condoms, oral pills, injectables and implants.

** Percentages may not add up to 100% for some clients were referred for more than one service

HTS – HIV Testing Services; VMMC – Voluntary Male Medical Circumcision;

STI – Sexually Transmitted Infections; SRH – Sexual and Reproductive Health

HIV – Human Immuno-deficiency Virus

For peer review only

For peer review only - http://bmjopen.bmj.com/site/about/guidelines.xhtml

Table 3: Factors associated with non-referral for HIV Testing Services among counselled young people in Bulawayo and Mount Darwin, Zimbabwe (October – December 2018)

Socio-demographic characteristics	Total counselled	Not referred for HTS N	(%)	Unadjusted RR	(95% CI))	Adjusted aRR	(95% CI)
**Total**	3370	2946	(100)				
**Age (years)**							
10 – 14	1242	1178	(94.8)	Ref		Ref	
15 – 19	1346	1134	(84.2)	**0.89**	**(0.82-0.96)**	**0.91**	**(0.83-0.99)**
20 – 24	782	634	(81.1)	**0.85**	**(0.78-0.94)**	0.92	(0.81-1.03)
**Sex**							
Male	2207	1937	(87.8)	1.01	(0.93-1.01)	0.98	(0.90-1.05)
Female	1163	1009	(86.8)	Ref		Ref	
**Marital Status**							
Single	3288	2889	(87.9)	Ref		Ref	
Married	77	52	(67.5)	0.77	(0.58-1.01)	0.88	(0.67-1.17)
Divorced or Separated	5	5	(100.	1.14	(0.47-2.74)	1.35	(0.58-3.25)
**School status**							
In-school	2334	2118	(90.7)	Ref		Ref	
Out of school	1036	828	(79.9)	**0.88**	**(0.81-0.95)**	0.95	(0.86-1.05)
**Counseling session**							
Individual	741	554	(74.8)	Ref		Ref	
Group	2629	2392	(91.0)	**1.22**	**(1.11-1.33)**	**1.16**	**(1.04-1.27)**
**Type of setting**							
Bulawayo (urban)	2160	1958	(90.6)	Ref		Ref	
Mount Darwin (rural)	1210	988	(81.7)	**0.90**	**(0.83-0.97)**	0.93	(0.85-1.01)
**Age of peer educator (years)[a]**							
15 - 19	751	673	(89.6)	Ref		Ref	
20 - 24	2619	2273	(86.8)	0.97	(0.89-1.05)	0.96	(0.85-1.03)
**Sex of peer educator[b]**							
Male	1624	1421	(87.5)	1.00	(0.93-1.07)	1.01	(0.93-1.08)
Female	1746	1525	(87.3)	Ref		Ref	

[a] Refers to the number of clients who were counselled by peer educators aged 15 – 19 and 20 - 24 years.

[b] Refers to the number of clients who were counselled by male and female peer educators.

HTS – HIV Testing Services; RR – Relative Risk; aRR – adjusted Relative Risk; CI – Confidence Interval

In bold – statistically significant at $p<0.05$

For peer review only

For peer review only - http://bmjopen.bmj.com/site/about/guidelines.xhtml

Table 4: Factors associated with non-referral for VMMC services among counselled young people in Bulawayo and Mount Darwin, Zimbabwe (October – December 2018)

Socio-demographic characteristics	Total counselled	Not referred N	(%)	Unadjusted RR	(95% CI))	Adjusted aRR	(95% CI)
**Total**	2207	92	(100)				
**Age (years)**							
10 – 14	944	28	(30.4)	Ref		Ref	
15 – 19	795	36	(45.5)	**1.50**	**(1.28-1.74)**	**1.59**	**(1.34-1.87)**
20 – 24	468	27	(57.9)	**1.90**	**(1.61-2.25)**	**1.83**	**(1.49-2.26)**
**Marital Status**							
Single	2179	90	(41.5)	Ref		Ref	
Married	27	15	(55.6)	1.34	(0.80-2.23)	1.31	(0.78-2.22)
Divorced or Separated	1	1	(100)	2.41	(0.34-17.1)	2.46	(0.34-1.77)
**School status**							
In-school	1613	60	(37.4)	Ref		Ref	
Out of school	594	31	(53.2)	**1.42**	**(1.24-1.63)**	1.09	(0.92-1.30)
**Counseling session**							
Individual	427	20	(48.2)	Ref		Ref	
Group	1780	71	(40.1)	**0.83**	**(0.71-0.97)**	0.86	(0.73-1.01)
**Type of setting**							
Bulawayo (urban)	1392	64	(46.3)	Ref		Ref	
Mount Darwin (rural)	815	27	(33.9)	**0.73**	**(0.64-0.84)**	**0.61**	**(0.52-0.71)**
**Age of peer educator (years)[a]**							
15 - 19	507	21	(42.8)	Ref		Ref	
20 - 24	1700	70	(41.4)	0.97	(0.83-1.13)	0.92	(0.78-1.08)
**Sex of peer educator[b]**							
Male	1159	44	(38.0)	0.83	**(0.73-0.94)**	**0.77**	**(0.67-0.80)**
Female	1048	48	(45.8)	Ref		Ref	

[a] Refers to the number of clients who were counselled by peer educators aged 15 – 19 and 20 - 24 years.

[b] Refers to the number of clients who were counselled by male and female peer educators.

VMMC – Voluntary Medical Male Circumcision; RR – Relative Risk; aRR – adjusted Relative Risk; CI – Confidence Interval

In bold – statistically significant at $p<0.05$

For peer review only

For peer review only - http://bmjopen.bmj.com/site/about/guidelines.xhtml

Table 5: Factors associated with non-receipt of VMMC services among referred young people in Bulawayo and Mount Darwin, Zimbabwe (October – December 2018)

Socio-demographic characteristics	Total referred	No receipt for VMMC services N	(%)	Unadjusted RR	(95% CI))	Adjusted aRR	(95% CI)
**Total**	1287	406	(100)				
**Age (years)**							
10 – 14	657	202	(30.7)	Ref		Ref	
15 – 19	433	136	(31.4)	1.02	(0.82-1.27)	1.24	(0.84-1.51)
20 – 24	197	68	(34.5)	1.12	(0.85-1.48)	1.29	(0.91-1.83)
**Marital Status**							
Single	1275	403	(31.6)	Ref		Ref	
Married	12	3	(25.0)	0.79	(0.25-2.46)	0.87	(0.28-2.74)
**School status**							
In-school	1009	328	(32.5)	Ref		Ref	
Out of school	278	78	(28.1)	0.86	(0.67-1.10)	0.80	(0.58-1.10)
**Counseling session**							
Individual	221	102	(46.2)	Ref		Ref	
Group	1066	304	(28.5)	**0.62**	**(0.49-0.77)**	**0.52**	**(0.41-0.66)**
**Type of setting**							
Bulawayo (urban)	748	284	(38.0)	Ref		Ref	
Mount Darwin (rural)	539	122	(22.6)	**0.60**	**(0.48-0.74)**	**0.52**	**(0.41-0.67)**
**Age of peer educator (years)[a]**							
15 - 19	290	67	(23.1)	Ref		Ref	
20 - 24	997	339	(34.0)	**1.47**	**(1.13-1.91)**	1.13	(0.84-1.51)
**Sex of peer educator[b]**							
Male	719	218	(30.3)	0.92	(0.75-1.11)	**0.76**	**(0.62-0.94)**
Female	568	188	(33.1)	Ref		Ref	

[a] Refers to the number of clients who were referred by peer educators aged 15 – 19 and 20 - 24 years.

[b] Refers to the number of clients who were referred by male and female peer educators.

VMMC – Voluntary Medical male Circumcision; RR – Relative Risk; aRR – adjusted Relative Risk; CI – Confidence Interval

In bold – statistically significant at $p<0.05$

For peer review only - http://bmjopen.bmj.com/site/about/guidelines.xhtml

Table 6: Factors associated with non-receipt of contraception services* among referred young people in Bulawayo and Mount Darwin, Zimbabwe (October – December 2018)

Socio-demographic characteristics	Total referred	No receipt for Contraception services		Unadjusted		Adjusted	
		N	(%)	RR	(95% CI))	aRR	(95% CI)
**Total**	452	89	(100%)				
**Age (years)**							
10 – 14	29	5	(17.2)	Ref		Ref	
15 – 19	175	30	(17.1)	0.99	(0.39-2.56)	0.93	(0.35-2.44)
20 – 24	248	54	(21.8)	1.26	(0.51-3.16)	1.42	(0.55-3.67)
**Sex**							
Male	246	44	(17.9)	0.82	(0.54-1.24)	0.73	(0.46-1.14)
Female	206	45	(21.8)	Ref		Ref	
**Marital Status**							
Single	399	82	(20.6)	Ref		Ref	
Married	48	4	(8.3)	0.41	(0.15-1.11)	**0.20**	**(0.07-0.58)**
Divorced or Separated	5	3	(60.0)	2.92	(0.92-9.24)	1.38	(0.42 -4.52)
**School status**							
In-school	116	30	(25.9)	Ref		Ref	
Out of school	336	59	(17.6)	0.68	(0.44-1.05)	0.83	(0.51-1.35)
**Counseling session**							
Individual	170	43	(25.3)	Ref		Ref	
Group	282	46	(16.3)	**0.64**	**(0.43-0.98)**	0.71	(0.46-1.11)
**Type of setting**							
Bulawayo (urban)	284	32	(11.3)	Ref		Ref	
Mount Darwin (rural)	168	57	(33.9)	**3.01**	**(1.95-4.64)**	**3.18**	**(1.93-5.22)**
**Age of peer educator (years)[a]**							
15 - 19	49	18	(36.7)	Ref		Ref	
20 - 24	403	71	(17.6)	0.48	(0.29-0.80)	1.00	(0.56-1.82)
**Sex of peer educator[b]**							
Male	180	26	(14.4)	0.62	(0.39-0.98)	0.81	(0.49-1.32)
Female	272	63	(23.2)	Ref		Ref	

[a] Refers to the number of clients who were referred by peer educators aged 15 – 19 and 20 - 24 years.

[b] Refers to the number of clients who were referred by male and female peer educators.

RR – Relative Risk; aRR – adjusted Relative Risk; CI – Confidence Interval

*Contraceptives included condoms, injectables, oral pills and implant hormonal contraceptives

In bold – statistically significant at $p<0.05$

For peer review only
For peer review only - http://bmjopen.bmj.com/site/about/guidelines.xhtml

Role of youth peer educators in the uptake of sexual and reproductive health services by adolescents (10-19 years) in two selected districts of Zimbabwe

Investigators and Institution

Aveneni Mangombe[1], Jaya Prasad Tripathy[2,3,4], Ajay MV Kumar[2,3,4], Bernard Madzima[1], Sinokuthemba Xaba[1], Phillip O Owiti[2,3,4], Mbazi Senkoro[5], Kudakwashe Takarinda[1], Margaret Nyandoro[1], Simbarashe Mabaya[6,7], Julie Sumelston[6,7], Talent Makoni[1], Talent Tapera[8], Nonhlanhla Zwangobani[9]

1. Ministry of Health and Child Care, Harare, Zimbabwe
2. International Union Against Tuberculosis and Lung Disease, Paris, France
3. International Union Against Tuberculosis and Lung Disease, South-East Asia Office, New Delhi, India
4. International Union Against Tuberculosis and Lung Disease (The Union), Paris, France; National Tuberculosis, Leprosy and Lung Disease Program, Nairobi, Kenya
5. National Institute for Medical Research – Muhimbili Centre, Dar es Salaam, Tanzania
6. World Health Organization, Harare Zimbabwe
7. World Health Organization, Geneva, Switzerland
8. Africaid, Harare, Zimbabwe
9. Zimbabwe National Family Planning Council, Harare, Zimbabwe

Corresponding Author:

Aveneni Mangombe

National Adolescent Sexual Reproductive Health Programme Coordinator,

Ministry of Health, Harare, Zimbabwe.

Email: mangombeaveh@gmail.com

Telephone: +263773302742

For peer review only - http://bmjopen.bmj.com/site/about/guidelines.xhtml

BACKGROUND

Human Immunodeficiency Virus (HIV) is a major public health problem globally, with 1.8 million new infections and 0.94 million deaths in 2017.(1) However, global commitment and sustained efforts have led to a decline in new HIV infections by 18% and AIDS-related deaths by 34% since 2010. Despite this, low rates of HIV diagnosis and treatment initiation among adolescents and young people continue to pose a significant challenge in the fight against the disease.(2)

Against a global decline in death rate due to AIDS-related illnesses, the rate of death among adolescents has been slower compared to adults, which is a concern worldwide.(2) Coverage of HIV testing and access to treatment remain significantly low among young people, especially in Sub-Saharan Africa.(2) Studies show low uptake of contraception, high rates of sexually transmitted infections (STIs), teenage pregnancy, adolescent suicide attempts, alcohol and drug use among this vulnerable age group.(2) Systematic reviews have also reported poor uptake of Adolescent Sexual and Reproductive Health (ASRH) services in different settings.(3)

Zimbabwe is one of the countries with the highest proportion of adolescents in the world, with a quarter of the population between the ages 10-19 years.(4) These adolescents face a myriad of challenges with respect to their sexual and reproductive health. Mirroring the global scenario, Zimbabwe Demographic Health Survey (ZDHS) 2015 reports high fertility rate among young girls (15-19 years), low coverage of HIV testing among 15-19 years old (46% and 35% ever tested among boys and girls respectively) and poor knowledge about HIV prevention methods.(5) This increases the vulnerability of adolescents to acquire HIV which is also highlighted in the National ASRH Strategy II: 2016-2020.(6)

To address these gaps in access and utilization of services for adolescents, the Zimbabwe ASRH programme was established in 2009. The National ASRH Strategy 2016 – 2020 defines an essential set of ASRH services to be delivered through four different settings: schools, tertiary colleges, community and health facilities. A recent review of the ASRH program highlighted several challenges and limitations including poor program coordination that has impacted service provision and access.(7)

For peer review only - http://bmjopen.bmj.com/site/about/guidelines.xhtml

The Ministry of Health and Child Care (MoHCC) also adopted voluntary medical male circumcision (VMMC) as an additional HIV prevention strategy in 2009 among male adolescents.(8,9) The common challenges affecting the adolescents, significant overlap of the interventions, as well as their mutually reinforcing nature justify the integration of VMMC and ASRH programmes to maximise the reach and service provision.

In line with this, the MoHCC with support from the World Health Organisation (WHO) commenced the pilot implementation of the VMMC - ASRH Linkages project in two jurisdictions: Bulawayo city and Mount Darwin district in 2016. Various models for integrating services were implemented in a range of settings including hospitals, clinics and other health facilities, youth corners, youth-friendly centres and the community. Within this project, the role of incentivized youth peer educators is integral in reaching out to the adolescents in the community and linking them to appropriate ASRH and VMMC services in designated facilities through a referral network.

Peer education has been found consistently to increase knowledge and improve attitudes and intentions. Two systematic reviews have demonstrated no to moderate effect of peer interventions in improving access to ASRH services suggesting further research. Chandra-Mouli et al. in a comprehensive review, concluded that, coordinated and complementary approaches are needed to improve the effectiveness of peer interventions and other ASRH programs.(10,11) The VMMC - ASRH linkage project is one such unique coordinated approach that seeks to expand the scope of work for youth peer educators in promoting SRH service uptake.

AIM:

In this context, we plan to conduct an operational research study to assess the role of youth peer educators in uptake of sexual and reproductive health (SRH) and voluntary male medical circumcision (VMMC) services among adolescents (10 – 19 years) within a pilot linkage project setting in Zimbabwe

For peer review only - http://bmjopen.bmj.com/site/about/guidelines.xhtml

OBJECTIVES:

Among adolescents (10-19 years) who were counselled by youth peer educators in Bulawayo city and Mt Darwin district in Zimbabwe during October-November 2018, to

- Describe their socio-demographic characteristics,
- Determine the number and proportion referred by peer educators and describe the services for which they were referred
- Determine the number and proportion among those referred who availed HIV testing, Family Planning, STI diagnosis and treatment and VMMC services, and assess the socio-demographic characteristics of the peer educators and the referred adolescents associated with it

METHODS:

Study Design:

This is a cohort study involving analysis of routinely collected data under the VMMC-ASRH linkage pilot project

Setting:

General setting: Zimbabwe is a landlocked low income country with a population of nearly 13.5 million as of 2017.(12) The 2012 National census indicated that adolescents (10-19 years) constitute 24% of the population(4). Zimbabwe has 10 provinces of which eight are rural and two are city provinces. Bulawayo is a city inhabited by 6,53,337 people.(4) It has 27 public health facilities including 3 central hospitals, one urban hospital and 23 clinics. Mount Darwin is a rural district with 2,12,725 people.(4) Health care in the district is provided by 19 public facilities which include one district hospital, one rural hospital, 17 primary care facilities (13 rural health centres and four clinics).

Specific setting:

ASRH Programme in Zimbabwe

In 2009, Zimbabwe devised the National ASRH strategy 2010-15 to promote adoption of safer SRH practices and increase availability, access and utilization of SRH services by young

For peer review only - http://bmjopen.bmj.com/site/about/guidelines.xhtml

people. The strategy outlined three key approaches for providing 'friendly' ASRH services: health facility, community, and school-based. The health facility approach required that every facility establish and equip special rooms called 'youth-friendly corners.' The community approach generally referred to 'community youth centres (CYCs) ', SRH drop-in centers or clubs. Finally, the school-based approach is essentially the provision of life skills education and counselling by both teachers and peer educators in schools.

The package of services included education and counselling on various topics, provision of contraceptives, pregnancy testing and management, STI diagnosis and management, post-rape care, HIV testing and counselling, post-abortion care, VMMC, recreational activities, referral and life and livelihood skills.(13)

Following this, the second National ASRH Strategy II: 2016 – 2020 was developed. This strategy is based largely on comprehensive sexuality education and WHO-standard based youth-friendly service provision, with a special focus on reducing new HIV infection, early marriages and adolescent fertility. Under this new strategy, ASRH is a package of interventions provided for those aged between 10 and 24 years delivered through schools and colleges (primarily comprehensive sexual health education), community based activities and public health facilities. The CYCs are primarily focused on recreational services by promoting youth development through sports, other recreational services, counselling and life skills training. The adolescents are registered for ASRH services at youth centres and referred to public health facilities in case they require any clinical services.

VMMC services

The MoHCC accepted VMMC as an additional HIV prevention strategy in 2009(14). At the inception of the programme, it targeted the 20 – 29 year olds and later on reached out to the infants and adolescent boys from 2014. The VMMC services are being provided at secondary and tertiary level hospitals, selected clinics and outreach camps by doctors and trained nurses. Clients are mobilized from the community by community based VMMC mobilizers and peer educators. Incentives are provided for mobilizers, for each client they

For peer review only - http://bmjopen.bmj.com/site/about/guidelines.xhtml

successfully refer to VMMC site. Zimbabwe plans to provide VMMC services to all infants upto 2 months and at least 80% of all males aged 10-29 years.

The VMMC-ASRH Linkages Project – "Smart LyncAges Project"

With support from the WHO, the Ministry of Health and Child Care commissioned an VMMC - ASRH Linkages project in Bulawayo city and Mount Darwin district in 2014.(9) The project is premised on the understanding that linking VMMC and ASRH programmes provide entry points for each other thereby improving access to and utilization of services. The implementation of the project began in 2016, following a series of planning meetings and development of the relevant VMMC- ASRH Linkages protocols. The project was branded ***"Smart LyncAges Project"*** during the course of implementation through a participatory process.

In Mount Darwin district, VMMC services are provided through the district hospital, whilst ASRH services are being provided at Mount Darwin Hospital, Mt Darwin and Dotito CYCs. In Bulawayo city, VMMC services are being provided through the Bulawayo Male Circumcision (MC) site and the Lobengula MC site (supported by an NGO called Population Services International (PSI)) and sometimes through outreach camps in the CYCs and clinics. The Bulawayo City Department of Housing also runs 15 CYCs which provide basic ASRH services through youth officers and peer educators, whilst the City health department's 22 clinics also provide SRH services.

The first phase of the project had limitations in data monitoring and reporting which preclude us from doing a cohort-wise monitoring of the beneficiaries. However, in the second phase which is planned for September - December 2018, the routine data collection and reporting formats have been revised to allow cohort-wise tracking of adolescents from the first point of contact to the point of service delivery to assess whether the beneficiaries have actually received the service or not.

Peer education intervention

In both Mount Darwin and Bulawayo, peer education is an integral component of the project. Peer educators (both males and females) are community based volunteers aged

For peer review only - http://bmjopen.bmj.com/site/about/guidelines.xhtml

18-24 years, selected through participatory community based platforms. They undergo a 7-day standard training on ASRH, using the National Standard ASRH Training Manual for Service Providers. Under this project, the peer educators additionally received an extra day to orient them on the project and their scope of work. They receive a USD 20 monthly allowance. They are supposed to be holders of at least 3 ordinary level qualifications and need to demonstrate ability to read and write. Each Community Youth Centre is expected to have a minimum of 5 peer educators at any particular time. Mount Darwin has 10 peer educators whilst Bulawayo city has a total of 75. They distribute condoms and provide counseling on sexual and reproductive health issues, education, career, drug abuse, relationship problems, etc. through individual and group approaches. Peer educators facilitated referrals to ASRH and VMMC sites using referral slips for services like diagnosis and management of STIs, HIV testing and counselling, VMMC, family planning and management of pregnancy. Peer educators are also expected to track the referred clients and in some cases accompany them to the service delivery points to ensure that they avail the service. Every month, each peer educator has a target of reaching 40 adolescent clients aged 10-24, two thirds being the 10-19 year olds.

Every peer educator maintains a ‘peer educator register’ where they document the socio-demographic details of the people who received counselling. They also record whether adolescents were referred and the type of services for which they were referred to. During referral, the peer educator fills a client referral form in triplicate: one copy is retained with the peer educator whilst the other two copies are given to the adolescent client to redeem at the referral service delivery point. After the client receives the services, the service provider at the referral centre fills details of the services provided to the client in both the copies of the referral form; one is retained by the referral centre whilst the other one is posted back to the referring peer educator or delivered in person. Every service delivery point also enters the client details into the primary registers for HTS, VMMC, Family Planning and STI diagnosis and treatment. After the peer educator receives the referral slip, s/he fills the column “availed service” in the peer educator register to denote that the client has received a particular service.

For peer review only - http://bmjopen.bmj.com/site/about/guidelines.xhtml

Study population:

All adolescents (10-19 years) who were counselled by youth peer educators during the period October – November 2018 period in project sites of Bulawayo city and Mount Darwin district will be included in the study. We expect around 3500 adolescents to be counselled during the study period.

Data variables, sources of data and data collection:

Data variables are listed according to study objectives:

Objective 1 (baseline socio-demographic characteristics): data variables include: age group (10-14, 15-19 years), sex, marital status, school status of adolescent clients (in/out); type of counselling session (group/individual). The source of data will be the Peer Educator registers.

Objective 2 (the number and proportion referred by peer educators and describe the services they were referred for): data variables include: referred (yes/no), type of service (HIV testing, VMMC, Family Planning, STI diagnosis and management and others). The source of data will be the Peer Educator registers.

Objective 3 (the number and proportion among those referred who availed the SRH services, and the socio-demographic characteristics of the peer educators and the referred adolescents associated with it): data variables include referred (yes/no), age and sex of peer educators; age group (10-14, 15-19 years), sex (male/female) and school status of adolescent clients (in/out), type of service availed (HIV Testing (yes/no), VMMC (yes/no), Family Planning (yes/no) and STI management (yes/no)). The source of data will be the Peer Educator registers. Client referral forms will be used to validate the responses in 10% of the referred clients.

Data will be collected into a structured proforma between January - March 2019 (See **Annexure 1**).

For peer review only - http://bmjopen.bmj.com/site/about/guidelines.xhtml

Analysis and statistics:

Double data entry, validation and analysis will be performed using EpiData software (Version 3.1 for entry and 2.2.2.180 for analysis; EpiData Association, Odense, Denmark). Proportions will be used to summarise referral, type of service for which referred and whether clients availed the service or not. Chi-square test (Fischer's Exact test wherever applicable) will be used to study the association of socio-demographic variables with availing service. The strength of association will be expressed using relative risk (RR) and adjusted RR with 95% CI using a log binomial regression method. Factors with a p value <0.2 in unadjusted analysis will be included in the regression model using STATA version 12.

Ethics approval

Ethics Issues: Permission for the study will be sought from the Ministry of Health and Child Care of Zimbabwe. Local ethics approval will be sought from Zimbabwe Medical Research Council of Zimbabwe (MRCZ). Ethics approval will also be sought from the Union Ethics Advisory Group, Paris, France.

Data confidentiality: Data will be entered in a designed format based on the information recorded in the registers. Names of clients will be used to trace patients and validate the data, but confidentiality will be maintained by keeping data collection forms securely in a lockable cabinet and the electronic data file will be kept in a password protected computer. Both data sets will be maintained securely for five years after completion of study. The names will be removed from the final dataset before analysis. Data will only be accessible to the study investigators.

Specific patient benefits: The results of this study will inform management about the ASRH – VMMC linkages referral practices by peer educators. This will result is strengthening the referral pathway for provision of integrated adolescent health services to the beneficiaries.

Community participation and benefits: The results will provide recommendations for strengthening the referral pathway, thereby maximizing service uptake and in the long run reduce incidence of STIs, HIV and unintended pregnancies among the general population as a whole.

For peer review only - http://bmjopen.bmj.com/site/about/guidelines.xhtml

Feedback and dissemination of results: The results of this study will be disseminated to the service providers and peer educators in Bulawayo and Mount Darwin and all supporting partners for the ASRH – VMMC Linkages project. The results will also be presented at national level through the National ASRH Coordination Forum, HIV Prevention partnership forums and international conferences. They will also be submitted in a peer reviewed journal.

Implications for policy and practice: There may be implications for policy and practice depending on the results of the study. It may provide additional evidence for the national scale up of the VMMC-ASRH linkages project.

Collaborative partnerships: These will be between the ASRH – VMMC Linkages Project partners (WHO, Harare, Zimbabwe, WHO, Geneva, Switzerland and Ministry of Health and Child Care, Zimbabwe) and The Union, Paris, France.

BUDGET:

Local project funds will be used for this study to train the peer educators on effective documentation, travel, stationary and overnight accommodation in Bulawayo and Mount Darwin. The submission of the paper to an open access journal will be supported by funds from The Union, Paris, France.

REFERENCES

1. Joint United Nations Programme on HIV/AIDS. Global HIV & AIDS statistics — 2018 fact sheet [Internet]. 2018 [cited 2018 Aug 10]. Available from: http://www.unaids.org/en/resources/fact-sheet

2. UNAIDS. Ending the AIDS epidemic for adolescents, with adolescents. 2016;36.

3. Denno DM, Hoopes AJ, Chandra-Mouli V. Effective Strategies to Provide Adolescent Sexual and Reproductive Health Services and to Increase Demand and Community Support. J Adolesc Heal. 2015 Jan;56(1):S22–41.

4. Zimbabwe National Statistics Agency. Zimbabwe Population Census 2012. Harare; 2013.

5. Zimbabwe National Statistics Agency and ICF International. Zimbabwe Demographic and Health Survey 2015: Final Report. Rockville, Maryland, USA; 2016.

6. Ministry of Health and Child Care Zimbabwe. National Adolescent and Youth Sexual

For peer review only

For peer review only - http://bmjopen.bmj.com/site/about/guidelines.xhtml

and Reproductive Health (ASRH) Strategy II: 2016 -2020. Harare; 2016.

7. Blum RW, Mmari K, Alfonso NY, Posner E. ASRH Strategic Plan Review for Zimbabwe. Harare, Zimbabwe; 2015.

8. Ministry of Health and Child Care Zimbabwe. Zimbabwe Policy Guidelines on Voluntary Medical Male Circumcision: Revised 2014. Harare; 2014.

9. Ministry of Health and Child Care Zimbabwe. Service Delivery Protocol for Strengthening Integration and Linkages between VMMC and ASRH Programmes in Zimbabwe. Harare; 2014.

10. Medley A, Kennedy C, O'Reilly K, Sweat M. Effectiveness of Peer Education Interventions for HIV Prevention in Developing Countries: A Systematic Review and Meta-Analysis. AIDS Educ Prev. 2009 Jun;21(3):181–206.

11. Chandra-Mouli V, Lane C, Wong S. What Does Not Work in Adolescent Sexual and Reproductive Health: A Review of Evidence on Interventions Commonly Accepted as Best Practices. Glob Heal Sci Pract. 2015 Aug 31;3(3):333–40.

12. Zimbabwe National Statistics Agency. Inter-Censal Demographic Survey, 2017. Harare; 2017.

13. Ministry of Health and Child Care Zimbabwe. National Adolescent Sexual and Reproductive Health Strategy: 2010 - 2015. Harare; 2009.

14. Ministry of Health and Child Care Zimbabwe. Accelerated Strategic and Operational Plan 2014 – 2018: Voluntary Medical Male Circumcision. Harare; 2014.

15. Ministry of Health and Child Care Zimbabwe. Zimbabwe Voluntary Medical Male Circumcision (VMMC) – Adolescent Sexual and Reproductive Health (ASRH) Linkages Pilot Project March 2016 – March 2017 (draft). Harare; 2018.

For peer review only - http://bmjopen.bmj.com/site/about/guidelines.xhtml

Annexure 1 Data Collection Proforma

Name of peer educator: ________________________________ Age: _______________ Sex (M/F):________

District: ___________________________________ Service delivery name: __________________________ Type of SDP: MC site/CYC

ID	Name of adolescent	Age group (1: 10-14, 2: 15-19)	Sex (M/F)	Marital status (use codes below)	Counselling (I/G)	School status (I/O)	Referred (Y/N)	21=FP		22=STIs		27=HTC		28=VMMC		Other services	
								R	A	R	A	R	A	R	A	R	A

MC: Male Circumcision; CYC: Community Youth Centre; Marital status: 1=Single and Never Married 2=Married 3=Divorced or Separated 4=Widowed ; R: Referred; A: Availed; Please write 'Y' which stands for Yes, if service is referred/availed and 'N' which stands for No if service is not referred/availed; M: Male; F: Female; I: Individual; G: Group; I: In; O: Out of school; HTS: HIV Testing and Counselling; FP: Family Planning; VMMC: Voluntary Medical Male Circumcision; STI: Sexually Transmitted Infections;

For peer review only

For peer review only - http://bmjopen.bmj.com/site/about/guidelines.xhtml

Reporting checklist for Aveneni Mangombe's cohort study.

Based on the STROBE cohort guidelines.

		Reporting Item	Page Number
**Title and abstract**			
Title	#1a	Indicate the study's design with a commonly used term in the title or the abstract	1
Abstract	#1b	Provide in the abstract an informative and balanced summary of what was done and what was found	3
**Introduction**			
Background / rationale	#2	Explain the scientific background and rationale for the investigation being reported	5
Objectives	#3	State specific objectives, including any prespecified hypotheses	6
**Methods**			
Study design	#4	Present key elements of study design early in the paper	7
Setting	#5	Describe the setting, locations, and relevant dates, including periods of recruitment, exposure, follow-up, and data collection	7
Eligibility criteria	#6a	Give the eligibility criteria, and the sources and methods of selection of participants. Describe methods of follow-up.	7
Eligibility criteria	#6b	For matched studies, give matching criteria and number of exposed and unexposed	7
Variables	#7	Clearly define all outcomes, exposures, predictors, potential confounders, and effect modifiers. Give diagnostic criteria, if applicable	7
Data sources / measurement	#8	For each variable of interest give sources of data and details of methods of assessment (measurement). Describe comparability of assessment methods if there is more than one group. Give information separately for for exposed and unexposed groups if applicable.	7
Bias	#9	Describe any efforts to address potential sources of bias	11
Study size	#10	Explain how the study size was arrived at	10
Quantitative	#11	Explain how quantitative variables were handled in the analyses. If applicable, describe which	11

For peer review only

variables		groupings were chosen, and why	
Statistical methods	#12a	Describe all statistical methods, including those used to control for confounding	11
Statistical methods	#12b	Describe any methods used to examine subgroups and interactions	N/A
Statistical methods	#12c	Explain how missing data were addressed	10
Statistical methods	#12d	If applicable, explain how loss to follow-up was addressed	N/A
Statistical methods	#12e	Describe any sensitivity analyses	N/A
**Results**			
Participants	#13a	Report numbers of individuals at each stage of study—eg numbers potentially eligible, examined for eligibility, confirmed eligible, included in the study, completing follow-up, and analysed. Give information separately for for exposed and unexposed groups if applicable.	12
Participants	#13b	Give reasons for non-participation at each stage	N/A
Participants	#13c	Consider use of a flow diagram	Table 1
Descriptive data	#14a	Give characteristics of study participants (eg demographic, clinical, social) and information on exposures and potential confounders. Give information separately for exposed and unexposed groups if applicable.	12
Descriptive data	#14b	Indicate number of participants with missing data for each variable of interest	12
Descriptive data	#14c	Summarise follow-up time (eg, average and total amount)	12
Outcome data	#15	Report numbers of outcome events or summary measures over time. Give information separately for exposed and unexposed groups if applicable.	12
Main results	#16a	Give unadjusted estimates and, if applicable, confounder-adjusted estimates and their precision (eg, 95% confidence interval). Make clear which confounders were adjusted for and why they were included	12 - 13
Main results	#16b	Report category boundaries when continuous variables were categorized	26
Main results	#16c	If relevant, consider translating estimates of relative risk into absolute risk for a meaningful time period	N/A
Other analyses	#17	Report other analyses done—e.g., analyses of subgroups and interactions, and sensitivity analyses	N/A
**Discussion**			

For peer review only

Key results	#18	Summarise key results with reference to study objectives	14
Limitations	#19	Discuss limitations of the study, taking into account sources of potential bias or imprecision. Discuss both direction and magnitude of any potential bias.	15
Interpretation	#20	Give a cautious overall interpretation considering objectives, limitations, multiplicity of analyses, results from similar studies, and other relevant evidence.	18
Generalisability	#21	Discuss the generalisability (external validity) of the study results	16
**Other Information**			
Funding	#22	Give the source of funding and the role of the funders for the present study and, if applicable, for the original study on which the present article is based	19

None The STROBE checklist is distributed under the terms of the Creative Commons Attribution License CC-BY. This checklist can be completed online using https://www.goodreports.org/, a tool made by the EQUATOR Network in collaboration with Penelope.ai

For peer review only

Does peer education go beyond giving reproductive health information? A cohort study in Bulawayo and Mount Darwin, Zimbabwe

Journal:	*BMJ Open*
Manuscript ID	bmjopen-2019-034436.R1
Article Type:	Original research
Date Submitted by the Author:	24-Dec-2019
Complete List of Authors:	Mangombe, Aveneni; Ministry of Health and Child Care, Family Health<br>Owiti, Philip; International Union Against Tuberculosis and Lung Disease (The Union); National Tuberculosis, Leprosy and Lung Disease Program<br>Madzima, Bernard; Ministry of Health and Child Care<br>Xaba, Sinokuthemba; Ministry of Health and Child Care, Harare Zimbabwe<br>Makoni, Talent; Ministry of Health and Child Care, AIDS and TB Unit ; World Health Organization , Zimbabwe, HIV Prevention<br>Takarinda, Kudakwashe ; Ministry of Health and Child Care<br>Timire, Collins ; International Union Against Tuberculosis and Lung Disease (The Union)<br>Chimwaza, Anesu; Ministry of Health and Child Care<br>Senkoro, Mbazi; National Institute for Medical Research – Muhimbili Centre<br>Mabaya, Simbarashe; World Health Organization Regional Office for Africa<br>Samuelson, Julia; World Health Organization<br>Ameyan, Wole ; World Health Organization<br>Tapera, Talent ; Africaid<br>Zwangobani, Nonhlanhla; Zimbabwe National Family Planning Council<br>Tripathy, Jaya; PGIMER, Community Medicine<br>Kumar, Ajay; International Union Against Tuberculosis and Lung Disease (The Union), South East Asia Office, Operational Research; International Union Against Tuberculosis and Lung Disease (The Union), Paris, France
**Primary Subject Heading**:	Sexual health
Secondary Subject Heading:	Health services research
Keywords:	Adolescents, Young people, voluntary medical male circumcision, adolescent sexual and reproductive health, SORT IT

SCHOLARONE™
Manuscripts

I, the Submitting Author has the right to grant and does grant on behalf of all authors of the Work (as defined in the below author licence), an exclusive licence and/or a non-exclusive licence for contributions from authors who are: i) UK Crown employees; ii) where BMJ has agreed a CC-BY licence shall apply, and/or iii) in accordance with the terms applicable for US Federal Government officers or employees acting as part of their official duties; on a worldwide, perpetual, irrevocable, royalty-free basis to BMJ Publishing Group Ltd ("BMJ") its licensees and where the relevant Journal is co-owned by BMJ to the co-owners of the Journal, to publish the Work in this journal and any other BMJ products and to exploit all rights, as set out in our licence.

The Submitting Author accepts and understands that any supply made under these terms is made by BMJ to the Submitting Author unless you are acting as an employee on behalf of your employer or a postgraduate student of an affiliated institution which is paying any applicable article publishing charge ("APC") for Open Access articles. Where the Submitting Author wishes to make the Work available on an Open Access basis (and intends to pay the relevant APC), the terms of reuse of such Open Access shall be governed by a Creative Commons licence – details of these licences and which Creative Commons licence will apply to this Work are set out in our licence referred to above.

Other than as permitted in any relevant BMJ Author's Self Archiving Policies, I confirm this Work has not been accepted for publication elsewhere, is not being considered for publication elsewhere and does not duplicate material already published. I confirm all authors consent to publication of this Work and authorise the granting of this licence.

For peer review only

Title: Does peer education go beyond giving reproductive health information? A cohort study in Bulawayo and Mount Darwin, Zimbabwe

Manuscript type: Original research article

Short Running Title: Enhanced reproductive health service uptake through peer education in Zimbabwe

Investigators and Affiliations

Aveneni Mangombe[1], Philip Owiti[2,3], Bernard Madzima[1], Sinokuthemba Xaba[1], Talent Makoni[1], Kudakwashe Collin Takarinda[1,2], Collins Timire[1,2], Anesu Chimwaza[1], Mbazi Senkoro[4], Simbarashe Mabaya[5], Julia Samuelson[6], Wole Ameyan[6], Talent Tapera[7], Nonhlanhla Zwangobani[8], Jaya Prasad Tripathy[9,10], Ajay MV Kumar[2,9,11]

1. Ministry of Health and Child Care, Harare, Zimbabwe

2. International Union Against Tuberculosis and Lung Disease (The Union), Paris, France;

3. National Tuberculosis, Leprosy and Lung Disease Program, Nairobi, Kenya

4. National Institute for Medical Research – Muhimbili Centre, Dar es Salaam, Tanzania

5. World Health Organization, Harare. Zimbabwe

6. World Health Organization, Geneva, Switzerland

7. Africaid, Harare, Zimbabwe

8. Zimbabwe National Family Planning Council, Harare, Zimbabwe

9. International Union Against Tuberculosis and Lung Disease, South-East Asia Office, New Delhi, India

10. All India Institute of Medical Sciences, Nagpur, India

11. Yenepoya Medical College, Yenepoya (Deemed to be University), Mangaluru, India

For peer review only - http://bmjopen.bmj.com/site/about/guidelines.xhtml

Corresponding Author:

Aveneni Mangombe, National Adolescent and Youth Sexual Reproductive Health Programme Coordinator, Ministry of Health and Child Care, Harare, Zimbabwe.

Email: mangombeaveh@gmail.com Telephone: +263773302742

Manuscript details

Word count: Abstract: 300 words; Main text: 3907

Number of tables: 6; **Number of figures**: 0

Number of references: 25

For peer review only

For peer review only - http://bmjopen.bmj.com/site/about/guidelines.xhtml

ABSTRACT

Objective: Peer education is an intervention within the voluntary medical male circumcision (VMMC)-adolescent sexual reproductive health (ASRH) linkages project in Bulawayo and Mount Darwin, Zimbabwe since 2016. Little is known if results extend beyond increasing knowledge. We therefore assessed extent of and factors affecting referral by peer educators and receipt of HIV testing services (HTS), contraception, management of sexually transmitted infections (STIs) and VMMC services by young people (10–24years) counselled.

Design: A cohort study involving all young people counselled by 95 peer educators during October-December 2018, through secondary analysis of routinely collected data.

Setting: All ASRH and VMMC sites in Mt Darwin and Bulawayo.

Participants: All young people counselled by 95 peer educators.

Outcome measures: Censor date for assessing receipt of services was 31 January 2019. Factors (clients' age, gender, marital & schooling status, counseling type, location and peer educators' age & gender) affecting non-referral and non-receipt of services (dependent variables) were assessed by log-binomial regression. Adjusted risk ratios (aRR) were calculated.

Results: Of the 3370 counselled (66% males), 65% were referred for at-least one service. 58% of males were referred for VMMC. Other services had 5-13% referrals. Non-referral for HTS decreased with clients' age(aRR:~0.9) but higher among group-counselled (aRR:1.16). Counseling by males (aRR:0.77) and rural location(aRR:0.61) reduced risks of non-referral for VMMC while age increased it(aRR≥1.59). Receipt of services was high (64-80%) except for STI referrals (39%). Group-counseling and rural location(aRR:~0.52) and male peer

For peer review only

For peer review only - http://bmjopen.bmj.com/site/about/guidelines.xhtml

educators(aRR:0.76) reduced risk of non-receipt of VMMC. Rural location increased risk of non-receipt of contraception(aRR:3.18) while marriage reduced it(aRR:0.20).

Conclusion: We found varying levels of referral ranging from 5.1%(STIs) to 58.3%(VMMC) but high levels of receipt of services. Type of counselling, peer educators' gender and location affected receipt of services. We recommend qualitative approaches to further understand reasons for non-referrals and non-receipt of services.

Key words: Adolescents, Young people, ASRH, Sexual and reproductive health, voluntary medical male circumcision, HIV Testing, Contraception, Sexually transmitted infections, Uptake and SORT IT.

Article Summary

Strengths and Limitations of this Study:

- This study included all the clients who were counselled by peer educators during the study period in the two project districts;
- It used routine programme data, thus making the findings a likely true reflection of the situation;
- The conduct and reporting of the study adhered to Strengthening the reporting of observational studies in epidemiology (STROBE) guidelines.
- The study couldn't exclude clients not in need of referral from the analysis due to lack of data from the peer educator registers in determining the reason for non-referral;
- Due to small sample size of those referred for STI diagnosis and treatment, the study lacked statistical power to carry out further analyses.

For peer review only

For peer review only - http://bmjopen.bmj.com/site/about/guidelines.xhtml

INTRODUCTION

Human Immunodeficiency Virus (HIV) still remains a major global public health concern, with 1.8 million new infections and 940,000 deaths reported in 2017.(1) Of concern is the slower decline of Acquired Immune Deficiency Syndrome (AIDS)-related illnesses and deaths among young people (10-24 years) compared to adults.(2) Coverage of HIV testing and access to treatment remain significantly low among young people, especially in sub-Saharan Africa.(2) Studies and systematic reviews have reported high rates of sexually transmitted infections (STIs), teenage pregnancy and suicide attempts, coupled with poor receipt of sexual and reproductive health (SRH) and HIV services among young people.(2,3)

Zimbabwe is a low-income country with a population of nearly 13.5 million as of 2017.(4) A third of the country's population are young people aged 10-24 years.(5) Mirroring the global scenario, the Zimbabwe Demographic Health Survey (ZDHS) 2015 reported a high fertility rate among young girls (15-19 years), low coverage of HIV testing among 15-19 years old (35%-46%) and low comprehensive knowledge on HIV.(6) Though SRH and HIV interventions focused on young people have been in existence for the past three decades, in Zimbabwe, the first 5-year strategic plan was developed in 2010. Among other things, this phase defined a minimum package of interventions that recognised the role of community-based youth peer educators in educating young people on SRH and HIV.

There is no universally agreed upon definition of peer education. We define it as a structured process of sharing of relevant information, values and behaviours among members of similar status, in an appropriate setting for both the educator and learner. Following a series of reviews, peer education has been noted to be an effective youth-led approach for influencing positive behavioural outcomes among beneficiaries, if given appropriate support systems and contextualized to different settings and needs of beneficiaries.(7,8) Peer education has been

For peer review only - http://bmjopen.bmj.com/site/about/guidelines.xhtml

established to be more effective in reaching out to key populations (for example, adolescents selling sex and who are sexually exploited) and delivering messages that are considered taboo to be delivered through schools, religious and family settings.(9) Integration of peer education interventions with holistic and well-coordinated interventions make it more effective towards improving health outcomes among beneficiaries in different contexts.(10,11)

In 2016, a project to link Voluntary Medical Male Circumcision (VMMC) with the Adolescent Sexual and Reproductive Health (ASRH) services (*"Smart LyncAges Project")* was started in Bulawayo city and Mount Darwin district. With a special focus on VMMC, the project targets both males and females for ASRH services. In 2017, the scope of the project was expanded to engage youth peer educators in promoting referral and receipt of SRH and VMMC services, informed by Michielsen et al's review(12).

Since the re-design, no study has been conducted to determine the effect of the new peer education model on the uptake of SRH and VMMC services, beyond just providing information to their peers. Within this background, we carried out a study to assess the referral and receipt of HIV testing services (HTS), contraception, diagnosis and treatment of sexually transmitted infections (STIs) and VMMC services among young people (10 – 24 years) counselled by the peer educators and their associated factors under the *"Smart LyncAges Project.*

For peer review only - http://bmjopen.bmj.com/site/about/guidelines.xhtml

METHODS

Study design and study population:

This was a cohort study involving young people aged 10-24 years (both males and females) counselled by 95 peer educators under the VMMC - ASRH linkages project in Bulawayo city and Mount Darwin district of Zimbabwe during October-December 2018. Secondary data routinely collected under the project on peer education and clients' service uptake were analysed.

Setting:

General setting

In 2009, Zimbabwe devised the National ASRH strategy 2010-15 to promote adoption of safer SRH practices and increase availability, access and utilization of SRH and HIV services by young people.(13) The strategy outlined three settings for providing 'friendly' SRH and HIV services: health facility, community and school-based. The health facility approach required every facility to establish and equip special rooms (youth-friendly corners). The community approach involved establishment of 'community youth centres (CYCs)' whilst the school-based approach focussed on the provision of life skills education and counselling mainly by teachers.

In 2015, an extensive review of the 2010-2015 interventions in Zimbabwe was conducted so as to inform the development of the National ASRH Strategy II: 2016-2020.(14) One of the review's conclusions acknowledged peer education as an important tool in ASRH programming, though with some modifications.(15) This strategy seeks to strengthen comprehensive sexuality education (CSE) and provision of quality-assured and adolescent-friendly services, delivered through schools and colleges, public health facilities and the

For peer review only

For peer review only - http://bmjopen.bmj.com/site/about/guidelines.xhtml

community. As part of expanding comprehensive sexuality education in both the in- and out-of-school settings, Zimbabwe aligned its tools and training materials with the 2018 United Nations Educational, Scientific and Cultural Organization (UNESCO) revised international technical guidance on sexuality education.(16) The minimum package of SRH services for young people includes contraception, STI diagnosis and treatment, HTS and integration of VMMC.

The VMMC - ASRH linkages project

The VMMC - ASRH linkages project has been implemented in Bulawayo and Mount Darwin, Zimbabwe since 2016. Bulawayo is an urban city with 27 public health facilities while Mount Darwin is a rural district with 19 public health facilities. In Mount Darwin, VMMC services are provided at the Mount Darwin Hospital, whilst diagnosis and treatment of STIs, contraception and HTS services are being provided at Mount Darwin Hospital and two CYCs (Mt Darwin and Dotito) supported through the Zimbabwe National Family Planning Council. In Bulawayo, VMMC services are provided at the Bulawayo Male Circumcision (MC) site and the Lobengula MC site and sometimes through outreach camps in the 15 CYCs and clinics. The CYCs in Bulawayo are primarily focused on imparting information and counselling services, edutainment services through films, drama and sports, library services and vocational and life skills training. All the service delivery points under this project were oriented on the VMMC – ASRH linkages service delivery protocols and ASRH. The VMMC – ASRH service delivery protocols clearly highlight the scope of work, reporting, referral, coordination and supervision mechanisms for peer educators. Demand creation for both SRH and VMMC services for young people relies heavily on community-based peer educators and VMMC mobilisers. Though the project has a special focus on males for VMMC, it targets both males and female for ASRH services.

For peer review only - http://bmjopen.bmj.com/site/about/guidelines.xhtml

In 2017, the *"Smart LyncAges Project"* project was redesigned and expanded the work of youth peer educators in promoting referral and receipt of SRH and VMMC services. The project also provided an updated training that introduced relevant tools for delivering the messages (distribution of information, education and communication materials and social media platforms such as facebook and WhatsApp), a clearly defined referral pathway (including referral forms, tracking and two-way feedback mechanism) and provision of adequate tools for documentation.

Peer education

Each CYC is expected to coordinate and supervise the work of 5 – 6 peer educators at any point in time. Peer educators are male and female volunteers aged 10-24 years, residing in the community and nominated by the young people in stakeholder community meetings. They must be able to read and write in English and the respective local language and should have passed at least 3 subjects at the end of secondary level (13th grade). Under the *Smart LyncAges project*, peer educators undergo a seven-day standard training, which also addresses the referral pathway. Each peer educator is attached to the nearest CYC and allocated a catchment area to cover. Peer educators are expected to contribute at least two hours a day for at least 3 days in a week to the project. Active peer educators receive a fixed monthly allowance of 15 USD, paid upon submission of daily and monthly summary reports of their activities.

While peer educators usually spend most of their time in the community conducting outreach sessions in pairs, they do sit in CYCs on a rotation basis to cater for the walk-in clients. At CYCs, considerations are made to ensure that at least one male and one female peer educator is available during the operating hours. Peer educators reach clients through both individual *(one-on-one)* and group counselling sessions (either individually or in pairs). The average duration of group counseling sessions is one hour whilst the size ranges from 15 to 25

For peer review only - http://bmjopen.bmj.com/site/about/guidelines.xhtml

participants. Depending on the sensitivity of the subject matter/topic, young people are usually grouped into 10 – 14 years, 15 – 19 years and 20 – 24 years clusters. Both peer educators and young people agree on the choice of places for group counseling sessions, such as recreation parks, play grounds, schools and community halls where young people usually congregate. In addition to providing information and counselling services, peer educators also distribute condoms to the clients. However, they refer clients for such when they run out of stock or are in settings (such as churches and schools) where condom distribution is prohibited.

Peer educators also facilitate referrals to ASRH and VMMC sites. The referral process includes providing information regarding location of the service delivery point, hours of operation, user fees (if any) and details of contact person (if available). They also complete referral forms in triplicate (one copy kept for records and two copies sent with the client to the destination service delivery point). One copy of the latter is retained at the service delivery point, while the other is given to the client who is expected to return it to the respective peer educator. The form retained at the service delivery point is sent to the respective CYC by post or hand-delivered. This system facilitates feedback on receipt of services by referred clients. Monthly, the peer educators are expected to track all the referred clients, through verification of redeemed referral forms, physical home visits or by telephone (where possible). In some cases, peer educators accompany clients to the service delivery points to ensure that they avail the service.

As part of documentation, each peer educator maintains individualized *"daily and monthly summary peer educator registers'* where they document the socio-demographic details of the clients who received counselling, the referrals and receipt of the services. Each service delivery point under the VMMC - ASRH Linkages project also uses primary registers for SRH and HIV services provision.

For peer review only - http://bmjopen.bmj.com/site/about/guidelines.xhtml

Data variables, sources of data and data collection:

A structured data collection tool was used to collect the following data variables: socio-demographic characteristics of clients (age, sex, marital status, location, schooling status, and type of counselling sessions undergone), the age and sex of their peer educators, whether referred or not, services referred for and if the clients received the services. The source of data was the peer educator registers. 'Referral' in this study meant that clients consented to receive some post-counseling services (such as HIV testing, VMMC, contraception or STI diagnosis and treatment) and were given a referral form. 'Receipt' of a service meant receiving/getting the services at a referral service delivery point by the young person who had been referred by peer educators (between October and December 2018) by end of January 2019.

Data Analysis and statistics:

Data entry and validation was performed using EpiData software (*v4.4.1.0, EpiData Association, Odense, Denmark*) while analyses were carried out using EpiData Analysis (*v2.2.2.186*) and STATA (*v14, StataCorp, College Station, Texas, USA*) softwares. Proportions were used to summarize referrals and receipt of services for those referred. There were four major services for which the clients were referred: VMMC (for males only), and HTS, STI diagnosis and treatment and contraception (for both males and females). Thus, the referral and receipt of these services were the key outcome variables for this study. Of these, factors associated with non-referral were assessed for HTS and VMMC services only. Factors associated with non-referrals for contraception and STI diagnosis and treatment were not analyzed as it was not possible to establish the appropriate denominator defining the eligibility for these services. Factors associated with non-receipt of services were assessed for VMMC and contraceptive use only. Non-receipt of HTS was not analyzed as HTS can also be provided as an opt-out provider-initiated testing and counseling (PITC) service at all

For peer review only - http://bmjopen.bmj.com/site/about/guidelines.xhtml

249 the service delivery points, irrespective of the reason for which the client was referred. HTS
250 was also a necessary service prior to conducting VMMC. Non-receipt of STI services
251 amongst those referred was not analyzed due to small sample size. The strength of
252 associations were initially expressed using unadjusted relative risks and then further
253 expressed using adjusted relative risks (aRR) and 95% confidence intervals, using log-
254 binomial regression methods. Two-sided $P<0.05$ were considered statistically significant.

For peer review only

For peer review only - http://bmjopen.bmj.com/site/about/guidelines.xhtml

RESULTS

There were 95 peer educators (52% males) in the study sites with a median age of 22 years (range: 15-24). A total of 3370 young people (2207 males and 1163 females) received counselling services from the peer educators (**Table 1**). Forty percent of young people were aged 15-19 years, with the majority being males (66%) and single (98%). Majority were still in school (69%) and received group counselling (78%).

Referrals and receipt of services

Table 2 shows the proportions of clients who were referred and those who received the services among those referred. Of the 3370 counselled young people, sixty five percent (75% of males and 47% of females) were referred for SRH services. Of those referred, 77% had been referred for only one service, with the rest being referred for two or more services. Seventy five percent (75% of males and 76% of females) of those referred received the services they had been referred for.

Of the males counselled, 58% were referred for VMMC services, of whom, 69% received the services. The other services for which the adolescents were referred for include HIV testing (13%), contraception (13%) and diagnosis and treatment of STIs (5%). Among the services referred for, the receipt for STI services was lowest (39%).

Factors associated with non-referrals for HTS and VMMC

Non-referral for HIV Testing Services (**Table 3**)

In multivariable analysis, only age of client and type of counseling were significantly associated with non-referral for HTS, holding other factors that influence HTS referral and receipt constant. Adolescents aged 15-19 years had a 9% (95% CI: 0.81-1.03) reduced risk of non-referral for HTS as compared to those aged 10-14 years old. Those who underwent

For peer review only - http://bmjopen.bmj.com/site/about/guidelines.xhtml

For peer review only

group counseling had 16% (95% CI: 1.04-1.27) increased risk of non-referral for HTS. Sex and marital status of the peer educators did not influence referral for HTS.

Non-referral for VMMC services (**Table 4**)

Compared to clients in the 10-14 year age group, those in the 15-19 year and 20-24 year age group had 59% (95% CI: 1.34-1.87) and 83% (95% CI: 1.49-2.26) increased risk of non-referral for VMMC, respectively. Clients in the rural district of Mount Darwin were at 39% (95% CI: 0.52-0.71) reduced risk of non-referral compared to those in the urban city of Bulawayo. Clients counseled on VMMC by male peer educators were at 23% (95% CI: 0.67-0.80) reduced risk for non-referral as compared to those counselled by female peer educators.

Factors associated with non-receipt for VMMC and contraception

Non-receipt of VMMC services (**Table 5**)

Clients referred by male peer educators had a 24% (95% CI: 0.62-0.94) lower risk of non-receipt of services than those referred by their female counterparts. Those referred from the rural district of Mount Darwin (aRR: 0.52, 95% CI: 0.41-0.67) and those referred through group counselling sessions (aRR: 0.52, 95% CI: 0.41-0.66) had significantly lower risk of non-receipt of VMMC services as compared to urban Bulawayo and individual counseling sessions, respectively. Age of the clients did not influence receipt of VMMC services.

Non-receipt of contraception services (**Table 6**)

Clients referred from the rural district had 3.2 times (95% CI: 1.93-5.22) higher risk of non-receipt of contraception services as compared to the urban-based counterparts. Married clients had 80% (95% CI: 0.07-0.58) reduced risk of not receiving contraception services than the single clients.

For peer review only - http://bmjopen.bmj.com/site/about/guidelines.xhtml

302 # DISCUSSION

303 This study, the first one assessing the role of the peer educators in the referral and receipt of
304 selected SRH and VMMC services among young people in Zimbabwe found relatively
305 moderate levels of referrals, particularly for VMMC services. A higher proportion of
306 females/girls were referred for each of the individual services as compared to males/boys.
307 However, overall more males were referred for any service compared to females likely due to
308 VMMC (for males only). However, for those referred, receipt of the services was high except
309 for STI diagnosis and treatment. Majority of the referred clients had been referred for just
310 one service, perhaps indicating limited counselling or reflecting the lack of need. The risk of
311 not being referred for HTS services decreased slightly with older age of clients, though for
312 VMMC services younger aged adolescents were more likely to be referred. Slightly
313 increased non-referral for HTS services was observed in clients who underwent group
314 counseling sessions as compared to individual sessions.

315 As regards receipt of services, generally more males received the services referred for as
316 compared to females. With regard to VMMC services, those counseled by male peer
317 educators were more likely to be referred and to receive, likewise to the clients in the rural
318 district. Rural setting increased risk of not receiving contraception services while being
319 married reduced this risk.

320 The low referral for diagnosis and treatment of STIs might have been influenced by: 1) lack
321 of confidence by peer educators in determining the need for referral. 2) limited self-reports of
322 STI-like symptoms. In any population, only a minority will have STI or STI - like symptoms
323 and referrals rates may be low. However, the low receipt of the diagnosis and treatment of
324 STIs services among the referred clients might have been due to the associated user fees
325 levied (mostly in council clinics and hospitals) in both the rural and urban settings.(17),(18)

For peer review only

For peer review only - http://bmjopen.bmj.com/site/about/guidelines.xhtml

Abolishing or largely subsidizing these costs, while maintaining or sustaining supply and diagnostics may improve receipt of services by young people.

Low risk perception among early adolescents has been established to be one of the barriers for referral and receipt of HTS services.(19) Other studies have also shown that demand for HTS services are reduced in adolescents who require parental/guardian consent, especially due to the perceived negative reactions from parents/guardians.(19) With Zimbabwe's age of consent for HTS at 16 years, this may explain the reduced non-referral for HTS as the client's age increases. Individualized or client-centered counseling has been established to be a more effective approach for HTS and post-test services than group counseling,(20) as demonstrated also by this study.

Group counseling sessions may have provided an opportunity for client peer influence on receipt of VMMC services. Counseling by male peer educators may also encourage openness among the male clients leading to more referral and receipt of VMMC services. As regards contraception, urban areas present more convenient service delivery points for accessing contraceptives than rural areas, explaining why non-receipt is lower in the urban setups. Myths on the association between modern contraception and future infertility(21),(22) may have resulted in the higher non-receipt rates of contraception among the adolescent girls referred. This belief may be more common in the rural setups and at times is propagated by other health workers.(22),(17)

Strengths: This study had several strengths. We included all the clients who were counselled by peer educators during the study period in the two project districts, which were the only districts implementing this intervention in the whole country. As we used routine programme data, the findings are likely a true reflection of the situation. The data was extracted into standard proformas by the principal investigator and trained data collectors – this enhanced

For peer review only

For peer review only - http://bmjopen.bmj.com/site/about/guidelines.xhtml

quality. Lastly, the conduct and reporting of the study adhered to Strengthening the Reporting of observational studies in epidemiology (STROBE) cohort reporting guidelines.(23)

Limitations: However, there were also limitations. First, due to deficiencies in documentation in the peer educator registers, we could not determine if the reason for non-referral (for VMMC or HTS) was related to 'need', that is, they had already received VMMC or HTS services before. We, therefore couldn't exclude clients not in need from the analysis. In certain circumstances, like referral for contraception and STI diagnosis and treatment, we could not perform further analyses as it was not possible to establish the appropriate denominator defining the eligibility for these services. Second, we were not able to establish exact reasons for non-referral and non-receipt of services. This requires qualitative and youth-led study approaches. Third, due to small sample size of those referred for STI diagnosis and treatment, we lacked statistical power to carry out further analyses. Many reviews on peer education have largely focused on assessing the designs or models of peer education without focusing on the outputs and the immediate positive outcomes of peer education in relation to receipt of diagnosis and treatment of STIs, HTS, contraception and VMMC services. Therefore, there were no study results to compare with. Whilst the results and conclusions of this study may be used in different peer education interventions, they cannot be generalised.

Implications: The study has the following implications:

- There is need for a review of peer education data collection tools to capture more client and peer educator data, for example, on details and quality of sessions and eligibility of clients for the various services in line with the recently adapted revised international guidelines on CSE in Zimbabwe.(24) The review will also need to be

For peer review only - http://bmjopen.bmj.com/site/about/guidelines.xhtml

followed with refresher training on the new concepts. This will help in determining actual output and factors associated with it.

- The project needs to consider integrating parent-child communication interventions into the VMMC – ASRH linkages project so as to mobilize parents to support the young people to access services (both psychosocially and financially). In the long run, this may also provide opportunities for home-based HTS and supervised HIV self-testing.
- While HTS services are better provided through individual sessions, VMMC services are better received when group counseling sessions are provided. There is thus need to focus on differentiated service provisions depending on circumstances, even as services are integrated.
- In view of the male peer educators having higher chances of effectively referring clients for VMMC, the peer education component needs to differentiate approaches which might include pairing of female peer educators with their male counterparts, to enhance the acceptability, confidence and capacity of female peer educators to mobilise, counsel and refer for VMMC services.
- There is a need to review and possibly abolish user fees attached to the diagnosis and treatment of STIs among the adolescents and young people in need of such services.
- Global standards on provision of quality health services to young people should be adopted and client satisfaction surveys prioritized to enhance quality service delivery.(17),(25) This will help contextualize peer education into service delivery and help understand the reasons for non-referral and non-receipt of services.

For peer review only

For peer review only - http://bmjopen.bmj.com/site/about/guidelines.xhtml

CONCLUSION

This study found varying levels of referrals ranging from 5.1% (STIs) to 58.3% (VMMC) but high levels of receipt of services among referred clients. Receipt of contraception, VMMC and HTS services was high among those referred. Factors affecting non-referral included age of client, sex of peer educator and type of counseling session (individual/group) while type of setting (rural/urban), age of client and sex of peer educators affected non-receipt of services. Peer education service differentiation based on gender and service type may further enhance uptake. Advocacy efforts for user fees removal and exemptions on SRH services for young people require accelerated investment to increase service uptake. There is also a need to review the output of the peer educator project with a view to enhancing the quality of care provided to the adolescents and young people. The study also recommends qualitative approaches to further understand reasons for non-referrals and non-receipt of services.

For peer review only

For peer review only - http://bmjopen.bmj.com/site/about/guidelines.xhtml

Acknowledgements

This research was conducted through the Structured Operational Research and Training Initiative (SORT IT), a global partnership led by the Special Programme for Research and Training in Tropical Diseases at the World Health Organization (WHO/TDR). The training model is based on a course developed jointly by the International Union Against Tuberculosis and Lung Disease (The Union) and Medécins sans Frontières (MSF). The specific SORT IT program which resulted in this publication was implemented by the Centre for Operational Research, The Union, Paris, France. Mentorship and the coordination/facilitation of this particular SORT IT workshop was provided through the Centre for Operational Research, The Union, Paris, France; the Department of Tuberculosis and HIV, The Union, Paris, France; the University of Washington, School of Public Health, Department of Global Health, Seattle, Washington, USA; National Institute for Medical Research, Muhimbili Centre, Dar es Salaam, Tanzania; and AIDS & TB Department, Ministry of Health & Child Care, Harare, Zimbabwe

Funding

The training course under which this study was conducted was funded by: the United Kingdom's Department for International Development (DFID); The Global Fund to Fight AIDS, Tuberculosis and Malaria (GFATM) and the World Health Organization. The funders had no role in study design, data collection and analysis, decision to publish, or preparation of the manuscript.

Author contributions

AM – the Principal Investigator

AM, TM, NZ, SM, SX and BM conceived the study.

AM, PO, AK, KCT, CT, JPT, NZ, SM, AC, MS and TT designed the study protocol.

For peer review only

For peer review only - http://bmjopen.bmj.com/site/about/guidelines.xhtml

AM, TM, NZ, SM, SX, BM, JS, WA, JPT and AC read and approved the protocol.

AM, TM collected the data.

AM, PO, AK, KCT, CT, JPT, NZ, SM, AC, MS and TT contributed to analyzing and interpreting the data.

AM, AK, PO, MS and TT drafted the manuscript.

AM, AK, PO, TM, NZ, SM, JS, WA, AC, MS, JPT, SX and BM critically revised the manuscript for intellectual content

All authors read and approved the final manuscript.

Disclosure Statement

The authors report no conflicts of interest

Ethics and Consent

Permission to undertake the study was granted by the Ministry of Health and Child Care. Ethics approval was obtained from the Medical Research Council of Zimbabwe (MRCZ/E/223) and the Ethics Advisory Group of the International Union Against Tuberculosis and Lung Disease, Paris, France (EAG number: 62/18). As this was a retrospective analysis of de-identified routine data, the need for individual client consent was waived by both ethics committees.

Data availability statement

The corresponding author can avail the data set on request without undue reservation.

Patient and Public Involvement

The principal investigator and enumerators had no interaction directly with the adolescents and young people whose records were reviewed. The registers were accessed from peer educators, community youth centre and health facility staff.

For peer review only

For peer review only - http://bmjopen.bmj.com/site/about/guidelines.xhtml

REFERENCES

1. Joint United Nations Programme on HIV/AIDS. Global HIV & AIDS statistics — 2018 fact sheet [Internet]. 2018 [cited 2018 Aug 10]. Available from: http://www.unaids.org/en/resources/fact-sheet

2. UNAIDS. Ending the AIDS epidemic for adolescents, with adolescents A practical guide to meaningfully engage adolescents in the AIDS response [Internet]. 2016 [cited 2019 May 9]. Available from: http://www.unaids.org/sites/default/files/media_asset/ending-AIDS-epidemic-adolescents_en.pdf

3. Denno DM, Hoopes AJ, Chandra-Mouli V. Effective Strategies to Provide Adolescent Sexual and Reproductive Health Services and to Increase Demand and Community Support. J Adolesc Heal [Internet]. 2015 Jan [cited 2018 Aug 10];56(1):S22–41. Available from: http://www.ncbi.nlm.nih.gov/pubmed/25528977

4. Zimbabwe National Statistics Agency. Inter-Censal Demographic Survey, 2017. Harare; 2017.

5. Zimbabwe National Statistics Agency. Zimbabwe Population Census 2012 [Internet]. Harare; 2013. Available from: http://www.zimstat.co.zw/dmdocuments/Census/CensusResults2012/National_Report.pdf

6. Zimbabwe National Statistics Agency and ICF International. Zimbabwe Demographic and Health Survey 2015: Final Report. Rockville, Maryland, USA; 2016.

7. Abdi F, Simbar M. The Peer Education Approach in Adolescents- Narrative Review Article. Iran J Public Health [Internet]. 2013 Nov [cited 2019 May 8];42(11):1200–6. Available from: http://www.ncbi.nlm.nih.gov/pubmed/26171331

8. Campbell C, Scott K, Mupambireyi Z, Nhamo M, Nyamukapa C, Skovdal M, et al. Community resistance to a peer education programme in Zimbabwe. BMC Health Serv Res [Internet]. 2014 Dec 19 [cited 2019 May 8];14(1):574. Available from: https://bmchealthservres.biomedcentral.com/articles/10.1186/s12913-014-0574-5

9. Hutton G, Wyss K, N'Diékhor Y. Prioritization of prevention activities to combat the spread of HIV/AIDS in resource constrained settings: a cost-effectiveness analysis from Chad, Central Africa. Int J Health Plann Manage [Internet]. 2003 Apr [cited 2019 May 9];18(2):117–36. Available from: http://www.ncbi.nlm.nih.gov/pubmed/12841152

10. Medley A, Kennedy C, O'Reilly K, Sweat M. Effectiveness of Peer Education Interventions for HIV Prevention in Developing Countries: A Systematic Review and Meta-Analysis. AIDS Educ Prev [Internet]. 2009 Jun [cited 2018 Aug 9];21(3):181–206. Available from: http://www.ncbi.nlm.nih.gov/pubmed/19519235

11. Chandra-Mouli V, Lane C, Wong S. What Does Not Work in Adolescent Sexual and Reproductive Health: A Review of Evidence on Interventions Commonly Accepted as Best Practices. Glob Heal Sci Pract [Internet]. 2015 Aug 31 [cited 2018 Aug 9];3(3):333–40. Available from: http://www.ghspjournal.org/lookup/doi/10.9745/GHSP-D-15-00126

For peer review only - http://bmjopen.bmj.com/site/about/guidelines.xhtml

12. Michielsen K, Chersich MF, Luchters S, De Koker P, Van Rossem R, Temmerman M. Effectiveness of HIV prevention for youth in sub-Saharan Africa: systematic review and meta-analysis of randomized and nonrandomized trials. AIDS [Internet]. 2010 May [cited 2019 May 8];24(8):1193–202. Available from: https://insights.ovid.com/crossref?an=00002030-201005150-00014

13. Ministry of Health and Child Care Zimbabwe. National Adolescent Sexual and Reproductive Health Strategy: 2010 - 2015. Harare; 2009.

14. Ministry of Health and Child Care Zimbabwe. National Adolescent and Youth Sexual and Reproductive Health (ASRH) Strategy II: 2016 -2020. Harare; 2016.

15. Blum RW, Mmari K, Alfonso NY, Posner E. ASRH Strategic Plan Review for Zimbabwe. Harare, Zimbabwe; 2015.

16. UNESCO. International technical guidance on sexuality education An evidence-informed approach [Internet]. 2018 [cited 2019 May 9]. Available from: http://www.unaids.org/sites/default/files/media_asset/ITGSE_en.pdf

17. Youth Engage. Mystery Client Visits in Zimbabwe Report: June 2017 - July 2018. 2018.

18. Ministry of Health and Child Care Zimbabwe. Zimbabwe Health Financing Strategy. 2017.

19. World Health Organisation. Adolescent HIV testing and counselling: a review of the literature [Internet]. World Health Organization; 2013 [cited 2019 May 8]. Available from: https://www.ncbi.nlm.nih.gov/books/NBK217943/

20. Sheon N. Theory and Practice of Client-Centered Counseling and Testing [Internet]. 2006. 2006 [cited 2019 May 8]. Available from: http://hivinsite.ucsf.edu/InSite?page=kb-07-01-04#S9.1X

21. Remez L, Woog V MM. Sexual and Reproductive Health Needs Of Adolescents in Zimbabwe. Brief, New York, Guttmacher Inst [Internet]. [cited 2019 May 8];2014 Serie(No 3):8. Available from: https://www.guttmacher.org/sites/default/files/report_pdf/ib-zimbabwe_0.pdf

22. Ngome E, Odimegwu C. The social context of adolescent women's use of modern contraceptives in Zimbabwe: a multilevel analysis. Reprod Health [Internet]. 2014 Dec 10 [cited 2019 May 8];11(1):64. Available from: http://www.ncbi.nlm.nih.gov/pubmed/25108444

23. von Elm E, Altman DG, Egger M, Pocock SJ, Gøtzsche PC, Vandenbroucke JP, et al. Strengthening the Reporting of Observational Studies in Epidemiology (STROBE) statement: guidelines for reporting observational studies. Lancet (London, England) [Internet]. 2007 Oct 20 [cited 2019 May 8];370(9596):1453–7. Available from: http://www.ncbi.nlm.nih.gov/pubmed/18064739

24. Herat J, Plesons M, Castle C, Babb J, Chandra-Mouli V. The revised international technical guidance on sexuality education - a powerful tool at an important crossroads for sexuality education [Internet]. Vol. 15, Reproductive Health. BioMed Central; 2018 Dec [cited 2019 May 9]. Available from: https://reproductive-health-journal.biomedcentral.com/articles/10.1186/s12978-018-0629-x

For peer review only - http://bmjopen.bmj.com/site/about/guidelines.xhtml

25. Ministry of Health and Child Care Zimbabwe. National Guidelines on Clinical Adolescent and Youth Friendly Sexual and Reproductive Health Services Provision (YFSP). 2016.

For peer review only

For peer review only - http://bmjopen.bmj.com/site/about/guidelines.xhtml

Table 1: Socio-demographic characteristics of young people counselled by peer educators in Bulawayo and Mount Darwin, Zimbabwe (October – December 2018):

Characteristics	N	(%)
Total	3370	(100)
Age of young people (years)		
10 – 14	1242	(36.9)
15 – 19	1346	(39.9)
20 – 24	782	(23.2)
Sex of young people		
Male	2207	(65.5)
Female	1163	(34.5)
Marital Status of young people		
Single	3288	(97.6)
Married	77	(2.3)
Divorced/Separated	5	(0.1)
Schooling status of young people		
In-school	2334	(69.3)
Out of school	1036	(30.7)
Type of counseling session received		
Individual	741	(22.0)
Group	2629	(78.0)
Type of Setting		
Bulawayo (Urban)	2160	(64.1)
Mount Darwin (Rural)	1210	(35.9)
Age of peer educator (years) [a]		
15 - 19	751	(22.3)
20 - 24	2619	(77.7)
Sex of peer educators		
Male	49	(51.6)
Female	46	(48.4)
Sex of peer educators reach [b]		
Male	1626	(48.2)
Female	1744	(51.8)

[a] Refers to the number of clients who were counselled by the peer educators aged 15 – 19 and 20 - 24 years.

[b] Refers to the number of clients who were counselled by male and female peer educators.

For peer review only - http://bmjopen.bmj.com/site/about/guidelines.xhtml

Table 2: Services for which counselled young people were referred for by peer educators and received in Bulawayo and Mount Darwin districts, Zimbabwe (October – December 2018)

Type of Service		Referred**		Received**	
		**n**	**(%)[a]**	**n**	**(%)[b]**
Referred for any service		2191	(65.0)	1645	(75.1)
	Females	544	(46.8)	413	(75.9)
	Males	1647	(74.6)	1232	(74.8)
HTS		424	(12.6)	271	(63.9)
	Females	154	(13.2)	88	(57.1)
	Males	270	(12.2)	183	(67.8)
VMMC *(among males only; n=2207)*		1287	(58.3)	881	(68.5)
Contraception*		452	(13.4)	363	(80.3)
	Females	206	(17.7)	161	(78.2)
	Males	246	(11.2)	202	(82.1)
STI diagnosis and treatment		171	(5.1)	67	(39.2)
	Females	87	(7.5)	28	(32.2)
	Males	84	(3.8)	39	(46.4)
Other SRH and HIV service		497	(14.7)	397	(79.9)
	Females	251	(21.6)	207	(82.5)
	Males	246	(11.2)	190	(77.2)

[a] – denominator is the total number counselled (N=3370), except for VMMC

[b] – denominator is the total number referred in the respective category

*Contraception refers to condoms, oral pills, injectables and implants.

** Percentages may not add up to 100% for some clients were referred for more than one service

For peer review only

For peer review only - http://bmjopen.bmj.com/site/about/guidelines.xhtml

HTS – HIV Testing Services; VMMC – Voluntary Male Medical Circumcision; STI – Sexually Transmitted Infections; SRH – Sexual and Reproductive Health HIV – Human Immuno-deficiency Virus

For peer review only

For peer review only - http://bmjopen.bmj.com/site/about/guidelines.xhtml

Table 3: Factors associated with non-referral for HIV Testing Services among counselled young people in Bulawayo and Mount Darwin, Zimbabwe (October – December 2018)

Socio-demographic characteristics	Total counselled	Not referred for HTS		Unadjusted		Adjusted	
		N	(%)	RR	(95% CI))	aRR	(95% CI)
**Total**	3370	2946	(100)				
**Age (years)**							
10 – 14	1242	1178	(94.8)	Ref		Ref	
15 – 19	1346	1134	(84.2)	**0.89**	**(0.82-0.96)**	**0.91**	**(0.83-0.99)**
20 – 24	782	634	(81.1)	**0.85**	**(0.78-0.94)**	0.92	(0.81-1.03)
**Sex**							
Male	2207	1937	(87.8)	1.01	(0.93-1.01)	0.98	(0.90-1.05)
Female	1163	1009	(86.8)	Ref		Ref	
**Marital Status**							
Single	3288	2889	(87.9)	Ref		Ref	
Married	77	52	(67.5)	0.77	(0.58-1.01)	0.88	(0.67-1.17)
Divorced or Separated	5	5	(100.	1.14	(0.47-2.74)	1.35	(0.58-3.25)
**School status**							
In-school	2334	2118	(90.7)	Ref		Ref	
Out of school	1036	828	(79.9)	**0.88**	**(0.81-0.95)**	0.95	(0.86-1.05)
**Counseling session**							
Individual	741	554	(74.8)	Ref		Ref	
Group	2629	2392	(91.0)	**1.22**	**(1.11-1.33)**	**1.16**	**(1.04-1.27)**
**Type of setting**							
Bulawayo (urban)	2160	1958	(90.6)	Ref		Ref	
Mount Darwin (rural)	1210	988	(81.7)	**0.90**	**(0.83-0.97)**	0.93	(0.85-1.01)
**Age of peer educator (years)[a]**							
15 - 19	751	673	(89.6)	Ref		Ref	
20 - 24	2619	2273	(86.8)	0.97	(0.89-1.05)	0.96	(0.85-1.03)
**Sex of peer educator[b]**							
Male	1624	1421	(87.5)	1.00	(0.93-1.07)	1.01	(0.93-1.08)
Female	1746	1525	(87.3)	Ref		Ref	

[a] Refers to the number of clients who were counselled by peer educators aged 15 – 19 and 20 - 24 years.

[b] Refers to the number of clients who were counselled by male and female peer educators.

HTS – HIV Testing Services; RR – Relative Risk; aRR – adjusted Relative Risk; CI – Confidence Interval

In bold – statistically significant at $p<0.05$

For peer review only

For peer review only - http://bmjopen.bmj.com/site/about/guidelines.xhtml

Table 4: Factors associated with non-referral for VMMC services among counselled young people in Bulawayo and Mount Darwin, Zimbabwe (October – December 2018)

Socio-demographic characteristics	Total counselled	Not referred N	(%)	Unadjusted RR	(95% CI))	Adjusted aRR	(95% CI)
**Total**	2207	92	(100)				
**Age (years)**							
10 – 14	944	28	(30.4)	Ref		Ref	
15 – 19	795	36	(45.5)	**1.50**	**(1.28-1.74)**	**1.59**	**(1.34-1.87)**
20 – 24	468	27	(57.9)	**1.90**	**(1.61-2.25)**	**1.83**	**(1.49-2.26)**
**Marital Status**							
Single	2179	90	(41.5)	Ref		Ref	
Married	27	15	(55.6)	1.34	(0.80-2.23)	1.31	(0.78-2.22)
Divorced or Separated	1	1	(100)	2.41	(0.34-17.1)	2.46	(0.34-1.77)
**School status**							
In-school	1613	60	(37.4)	Ref		Ref	
Out of school	594	31	(53.2)	**1.42**	**(1.24-1.63)**	1.09	(0.92-1.30)
**Counseling session**							
Individual	427	20	(48.2)	Ref		Ref	
Group	1780	71	(40.1)	**0.83**	**(0.71-0.97)**	0.86	(0.73-1.01)
**Type of setting**							
Bulawayo (urban)	1392	64	(46.3)	Ref		Ref	
Mount Darwin (rural)	815	27	(33.9)	**0.73**	**(0.64-0.84)**	**0.61**	**(0.52-0.71)**
**Age of peer educator (years)[a]**							
15 - 19	507	21	(42.8)	Ref		Ref	
20 - 24	1700	70	(41.4)	0.97	(0.83-1.13)	0.92	(0.78-1.08)
**Sex of peer educator[b]**							
Male	1159	44	(38.0)	0.83	**(0.73-0.94)**	**0.77**	**(0.67-0.80)**
Female	1048	48	(45.8)	Ref		Ref	

[a] Refers to the number of clients who were counselled by peer educators aged 15 – 19 and 20 - 24 years.

[b] Refers to the number of clients who were counselled by male and female peer educators.

VMMC – Voluntary Medical Male Circumcision; RR – Relative Risk; aRR – adjusted Relative Risk; CI – Confidence Interval

In bold – statistically significant at $p<0.05$

For peer review only

For peer review only - http://bmjopen.bmj.com/site/about/guidelines.xhtml

608 **Table 5: Factors associated with non-receipt of VMMC services among referred young**
609 **people in Bulawayo and Mount Darwin, Zimbabwe (October – December 2018)**

Socio-demographic characteristics	Total referred	No receipt for VMMC services N	(%)	Unadjusted RR	(95% CI))	Adjusted aRR	(95% CI)
**Total**	1287	406	(100)				
**Age (years)**							
10 – 14	657	202	(30.7)	Ref		Ref	
15 – 19	433	136	(31.4)	1.02	(0.82-1.27)	1.24	(0.84-1.51)
20 – 24	197	68	(34.5)	1.12	(0.85-1.48)	1.29	(0.91-1.83)
**Marital Status**							
Single	1275	403	(31.6)	Ref		Ref	
Married	12	3	(25.0)	0.79	(0.25-2.46)	0.87	(0.28-2.74)
**School status**							
In-school	1009	328	(32.5)	Ref		Ref	
Out of school	278	78	(28.1)	0.86	(0.67-1.10)	0.80	(0.58-1.10)
**Counseling session**							
Individual	221	102	(46.2)	Ref		Ref	
Group	1066	304	(28.5)	**0.62**	**(0.49-0.77)**	**0.52**	**(0.41-0.66)**
**Type of setting**							
Bulawayo (urban)	748	284	(38.0)	Ref		Ref	
Mount Darwin (rural)	539	122	(22.6)	**0.60**	**(0.48-0.74)**	**0.52**	**(0.41-0.67)**
**Age of peer educator (years)[a]**							
15 - 19	290	67	(23.1)	Ref		Ref	
20 - 24	997	339	(34.0)	**1.47**	**(1.13-1.91)**	1.13	(0.84-1.51)
**Sex of peer educator[b]**							
Male	719	218	(30.3)	0.92	(0.75-1.11)	**0.76**	**(0.62-0.94)**
Female	568	188	(33.1)	Ref		Ref	

610

611 [a] Refers to the number of clients who were referred by peer educators aged 15 – 19 and 20 -
612 24 years.

613 [b] Refers to the number of clients who were referred by male and female peer educators.

614 VMMC – Voluntary Medical male Circumcision; RR – Relative Risk; aRR – adjusted
615 Relative Risk; CI – Confidence Interval

616 **In bold** – statistically significant at $p<0.05$

617

618

For peer review only - http://bmjopen.bmj.com/site/about/guidelines.xhtml

Table 6: Factors associated with non-receipt of contraception services* among referred young people in Bulawayo and Mount Darwin, Zimbabwe (October – December 2018)

Socio-demographic characteristics	Total referred	No receipt for Contraception services		Unadjusted		Adjusted	
		N	(%)	RR	(95% CI))	aRR	(95% CI)
**Total**	452	89	(100%)				
**Age (years)**							
10 – 14	29	5	(17.2)	Ref		Ref	
15 – 19	175	30	(17.1)	0.99	(0.39-2.56)	0.93	(0.35-2.44)
20 – 24	248	54	(21.8)	1.26	(0.51-3.16)	1.42	(0.55-3.67)
**Sex**							
Male	246	44	(17.9)	0.82	(0.54-1.24)	0.73	(0.46-1.14)
Female	206	45	(21.8)	Ref		Ref	
**Marital Status**							
Single	399	82	(20.6)	Ref		Ref	
Married	48	4	(8.3)	0.41	(0.15-1.11)	**0.20**	**(0.07-0.58)**
Divorced or Separated	5	3	(60.0)	2.92	(0.92-9.24)	1.38	(0.42 -4.52)
**School status**							
In-school	116	30	(25.9)	Ref		Ref	
Out of school	336	59	(17.6)	0.68	(0.44-1.05)	0.83	(0.51-1.35)
**Counseling session**							
Individual	170	43	(25.3)	Ref		Ref	
Group	282	46	(16.3)	**0.64**	**(0.43-0.98)**	0.71	(0.46-1.11)
**Type of setting**							
Bulawayo (urban)	284	32	(11.3)	Ref		Ref	
Mount Darwin (rural)	168	57	(33.9)	**3.01**	**(1.95-4.64)**	**3.18**	**(1.93-5.22)**
**Age of peer educator (years)[a]**							
15 - 19	49	18	(36.7)	Ref		Ref	
20 - 24	403	71	(17.6)	0.48	(0.29-0.80)	1.00	(0.56-1.82)
**Sex of peer educator[b]**							
Male	180	26	(14.4)	0.62	(0.39-0.98)	0.81	(0.49-1.32)
Female	272	63	(23.2)	Ref		Ref	

[a] Refers to the number of clients who were referred by peer educators aged 15 – 19 and 20 - 24 years.

[b] Refers to the number of clients who were referred by male and female peer educators.

RR – Relative Risk; aRR – adjusted Relative Risk; CI – Confidence Interval

*Contraceptives included condoms, injectables, oral pills and implant hormonal contraceptives

In bold – statistically significant at p<0.05

For peer review only - http://bmjopen.bmj.com/site/about/guidelines.xhtml

For peer review only
